# Insights on Postimplementation and Delivery of Telemedicine in Remote Areas of Rajasthan: Qualitative Study

**DOI:** 10.2196/93146

**Published:** 2026-10-16

**Authors:** Ramesh Kumar Huda, Rahul Singh Chowhan, Sumit Kalra, Ramesh Kumar Sangwan, Mukti Khetan

**Affiliations:** 1 National Institute for Implementation Research on Non-Communicable Diseases, New Pali Road, Bhagat ki Kothi, Jodhpur, Rajasthan, 342005, India; 2 Indian Institute of Technology Jodhpur, Jodhpur, Rajasthan, India

**Keywords:** medical informatics, telemedicine, implementation research, patient perspectives, digital health, rural health care

## Abstract

**Background:**

Telemedicine has emerged as a critical strategy to address health care access gaps in hard-to-reach areas, particularly in resource-constrained settings. While telemedicine services have expanded across India, evidence on post-implementation experiences, specifically regarding the integration of portable diagnostic tools, and the assisting role of frontline health care workers, remains limited. Understanding these sociotechnical dynamics is essential for optimizing digital health interventions in remote areas.

**Objective:**

This study aimed to explore perceptions, experiences, and implementation processes of an assisted telemedicine model where frontline health care workers facilitate real-time, doorstep patient-assisted-to-doctor consultations using portable diagnostics and a customized portal among health care workers and patients in hard-to-reach villages of Rajasthan, India.

**Methods:**

A qualitative descriptive study was conducted in 5 remote villages of Balotra district, Rajasthan, between December 2025 and January 2026, following a 10-month telemedicine intervention. In-depth, semistructured interviews were conducted with 28 participants, including medical officers (n=3, 10.71%), nurses, and accredited social health activists or auxiliary nurse midwives (n=10, 35.71%), and patients (n=15, 53.57%). Cadre-specific semistructured interview guides were developed using the Consolidated Framework for Implementation Research and Technology Acceptance Model. Interviews were analyzed using a reflexive thematic analysis approach, incorporating both inductive and deductive coding approaches in NVivo 15 (Lumivero). Triangulation across participant groups and analytic perspectives was used to enhance credibility and trustworthiness.

**Results:**

The analysis revealed that the assisted telemedicine model fundamentally reconfigured treatment-seeking behavior by providing doorstep care, significantly reducing travel burdens, and out-of-pocket expenditures. Frontline health care workers acted as crucial sociotechnical mediators, bridging the digital divide and fostering patient trust, particularly among women and older adults. Health care providers reported increased clinical confidence and workflow efficiency, supported by real-time vital monitoring and cross-verification via digital chatbots. However, implementation was challenged by intermittent network connectivity, diagnostic limitations for conditions requiring physical palpation, and occasional stock-outs of essential medicines. Despite these barriers, the gradual acceptance of the system was heavily facilitated by the continuous engagement and cultural mediation of local health workers.

**Conclusions:**

Telemedicine was perceived as a feasible and acceptable model for extending primary health care services in hard-to-reach areas, provided it is embedded within an assisted model supported by trained frontline workers and responsive technical systems. The findings emphasize the need to shift health care policy toward mediated-digital models, formally recognizing community health workers as digital health navigators. Future scale-up must prioritize strengthening rural digital infrastructure and ensuring a reliable supply chain for essential medicines to sustain long-term engagement and health equity.

## Introduction

Telemedicine has emerged as a critical tool for bridging health care access gaps, particularly in hard-to-reach areas with limited medical resources. Over the past two decades, it has evolved from experimental pilot projects into a core component of modern health delivery, extending specialist and primary care to remote populations [1]. This technology uses information and communication technologies to deliver health care services remotely, thereby enhancing accessibility and improving patient outcomes in remote areas [2]. It significantly reduces patient transportation costs and improves the efficiency of medical services in areas with scarce medical resources [3]. Nevertheless, the adoption of telemedicine worldwide remains uneven, with infrastructural, organizational, and regulatory barriers consistently limiting its scale-up in resource-constrained settings [4]. Telemedicine can lower overall health care costs by streamlining service delivery and minimizing the need for physical consultations. Moreover, it can empower individuals in these regions, who often face greater challenges in accessing health care, by improving inclusivity and participation in health care services [5,6].

The integration of telemedicine is especially pertinent in lower-middle-income countries such as India, where vast populations and significant geographical distances between urban centers and rural towns create substantial barriers to accessing specialized health care services. In addition, especially in regions such as Rajasthan, where geographical barriers and a sparse distribution of health care facilities often hinder timely access to essential medical care, telemedicine can promote equitable health care by extending specialist consultations and ongoing care to remote populations. Moreover, telemedicine implementation in Health and Wellness Centers facilitates consultation, screening, and remote monitoring, enabling individuals to connect with health care providers and receive medical advice. This is particularly beneficial for patients with chronic conditions, enabling continuous monitoring and timely interventions without extensive travel [7,8].

To contextualize this implementation, India’s national telemedicine initiatives, such as the eSanjeevani platform (developed by the Centre for Development of Advanced Computing, Ministry of Health and Family Welfare, Government of India), have largely focused on facility-based models (eg, doctor-to-doctor consultations at Health and Wellness Centres or subcenters) to bridge the urban-rural health care divide [9]. While these facility-based models address specialist shortages, they still require patients to travel to a local health facility, which remains a barrier in geographically isolated terrains. The intervention evaluated in this study represents a distinct piloting and evidence generation stage of an adapted mobile health model [8]. Specifically, it introduces an assisted telemedicine model that extends care beyond the subcenter level directly to the patient’s doorstep. In this workflow, frontline health care workers (accredited social health activists [ASHAs], auxiliary nurse midwives [ANMs], and nurses) conduct home visits or respond to patient calls, capture real-time vital parameters using portable digital diagnostic tools, and upload the data to a customized telemedicine portal. The health worker then facilitates a live video consultation between the patient and a remote medical officer, who generates an e-prescription that the health worker downloads and delivers to the patient. The added value of this specific intervention lies in its mobility and real-time diagnostic capability, effectively overcoming the geographical barriers inherent in standard facility-based models by bringing the digital health hub directly to the patient’s home [10,11]. This implementation-focused perspective is consistent with iCHECK-DH (Guidelines and Checklist for the Reporting on Digital Health Implementations; Checklist 1), which emphasizes documenting the contextual factors, implementation processes, and adaptations that shape digital health delivery in real-world settings [12].

This study aimed to address the gap by presenting in-depth qualitative findings from Balotra district of Rajasthan, which marks its geographical remoteness and limited health infrastructure on the post-implementation delivery of telemedicine. The insights on perception of acceptability, operational challenges, and actionable recommendations have been drawn from interviews with ASHAs, ANMs, nursing officers (NOs), medical officers (MOs), and patients who used the telemedicine facilities.

## Methods

### Study Design

A qualitative descriptive study design was used to understand the experiences and perceptions of frontline health care workers and patients involved in the implementation and delivery of telemedicine services in hard-to-reach areas of Balotra district of Rajasthan. The study used in-depth interviews (IDIs) to document lived experiences, perceived benefits, implementation challenges, acceptability, and recommendations for improvement among those who used telemedicine services during the intervention group’s implementation phase. This manuscript is reported in accordance with the COREQ (Consolidated Criteria for Reporting Qualitative Research) checklist (Checklist 2). Additionally, the reporting of the digital health implementation context, intervention characteristics, and structural barriers was guided by the iCHECK-DH mentioned in Checklist 1 [12].

### Study Setting

The study was conducted in 5 randomly chosen hard-to-reach areas of the Balotra district of Rajasthan between December 2025 and January 2026, where health care is severely constrained due to a lack of proper health care accessibility, geographical isolation, limited access to specialist care, a health care worker shortage, and minimal existing health care infrastructure. These areas were facilitated with telemedicine services, in which teleconsultations were operational from 8 AM to 5 PM for 5 working days, supported by trained frontline health care workers.

### Study Context

As part of the telemedicine intervention (parent project), a team of frontline health care workers, ASHAs or ANMs, and nurses were trained to operate digital diagnostic devices and a customized telemedicine portal and were deployed with medical kits and smartphones to facilitate teleconsultations with doctors. Five teams were assigned to the 5 randomly selected villages, and their contact information was shared across the community through an initial door-to-door survey. During operational hours, frontline workers visited patients at their doorsteps or patients visited them, measured vital parameters such as blood pressure (BP), oxygen saturation (SpO_₂_), and heart rate, conducted electrocardiography (ECG) if required, and provided real-time video consultations with the MOs sitting remotely. The MOs used patients’ data and interactions with patients to provide medical advice, e-prescriptions, and referrals, as needed. The telemedicine portal was developed on the MERN stack (MongoDB, Express, React, and Node.js) with WebRTC integration to allow secure transmission and storage of patient data, live e-prescription generation, and follow-up management.

### Study Participants

The study included 28 participants from multiple stakeholder groups who were directly involved in or had experience with telemedicine services during the intervention period, enabling a comprehensive understanding of telemedicine implementation and delivery in hard-to-reach areas. Purposive sampling was used to recruit both frontline health care workers and patients to ensure representation of diverse experiences and perspectives. Frontline health care workers (n=13) were purposively recruited through their respective primary health centers and project headquarters and comprised 5 (38.5%) ASHAs or ANMs, 5 (38.5%) nurses, and 3 (23.1%) MOs. Patients were purposively selected from the telemedicine intervention registry (n=4401) with assistance from local ASHA workers, who identified individuals with varied demographic characteristics, frequencies of telemedicine use, health conditions, and levels of engagement with the system. Fifteen patients, representing both genders and different age groups, were selected, with 3 participants recruited from each of the 5 intervention villages. This purposive selection ensured variation in participants’ experiences, symptoms, and patterns of telemedicine use, thereby providing diverse perspectives on the implementation and use of telemedicine services.

### Data Collection

Qualitative data were collected through face-to-face IDIs conducted post-implementation of the intervention project for 10 months within the intervention group villages. RS Chowhan, RKH, and project staff, holding ME, PhD, and MHA, respectively, conducted the IDIs. All received training in qualitative research methods, including thematic analysis and conducting IDIs, at Indian Council of Medical Research (ICMR)-National Institute for Implementation Research on Non-Communicable Diseases (NIIRNCD), Jodhpur. Separate semistructured interview guides were developed for ASHAs, ANMs, or nurses, MOs, and patients based on the Consolidated Framework for Implementation Research (CFIR) and technology acceptance model (TAM) frameworks after a literature review and through multiple rounds of discussion with the research team and domain experts to ensure questionnaire alignment with study objectives and to reflect differences in roles, experiences, and interaction with the telemedicine system.

Trained qualitative researchers with no prior relationship with participants conducted interviews in the local language (Hindi or Marwari). Each interview lasted approximately 30 to 35 minutes and was audio recorded with written consent in a quiet, private space, ensuring participants’ comfort, anonymity, and confidentiality. Semistructured interview guides for patients, MOs, and nurses were used to facilitate data collection (Multimedia Appendix 1).

### Data Analysis

The study was grounded in a critical realist epistemological perspective, acknowledging the objective realities of the rural health care infrastructure while recognizing that participants’ experiences and perceptions of the telemedicine system are socially constructed and context dependent. The audio recordings were transcribed into English by bilingual researchers and verified by the principal investigator for consistency, accuracy, and data integrity, without distorting participant intent throughout the translation process. Transcribed files and audio recordings were securely stored in password-protected folders. Data saturation was achieved when no new themes were obtained. The thematic analysis incorporated both deductive (literature-driven themes) and inductive (field-driven themes) coding approaches as described by Braun and Clarke using NVivo 15 software (Lumivero) [13]. This study used an integrated theoretical approach combining the CFIR and the TAM. CFIR guided the deductive analysis of multilevel implementation determinants, while TAM served as an interpretive framework to explore individual perceptions of telemedicine, including ease of use, usefulness, and intention to use.

Thematic saturation was assessed concurrently during data collection and analysis by the qualitative research team (RS Chowhan and RK). Saturation was assessed independently for each of the 3 stakeholder groups (MOs, frontline health care workers, and patients) to ensure diverse perspectives were adequately captured. Regarding the MOs (n=3, 10.71%), the intervention specifically deployed exactly 3 (10.71%) MOs to cover the 5 remote villages. Therefore, n=3 represents a complete census of the MO cadre involved in this specific pilot, not a sample. Their interviews were extensive (30‐40 min), and thematic saturation for their specific operational context was achieved within this bounded group. For frontline health care workers (n=10) and patients (n=15), recruitment continued until no new themes or codes emerged from successive interviews.

Triangulation was applied during data analysis to enhance credibility. Data source triangulation was achieved by comparing perspectives across MOs, frontline health care workers, and patients involved in the telemedicine intervention. Investigator triangulation was ensured through the involvement of multiple trained researchers in data collection and coding, with iterative discussions to reach consensus. Theoretical triangulation was further applied by integrating CFIR to examine implementation contexts and processes, alongside TAM to interpret individual perceptions of telemedicine use. Rigor and trustworthiness criteria, as described by Lincoln and Guba [14], were applied to enhance credibility, dependability, confirmability, and contextual transferability through data saturation, systematic coding, the use of verbatim quotations, and detailed descriptions of the study setting and participants.

In alignment with the COREQ checklist, the research team maintained reflexive journals during data collection and analysis to document personal assumptions and biases regarding digital health interventions. Field notes were taken during and immediately after each interview to capture nonverbal cues, environmental context, and initial analytical memos. There were no refusals or dropouts among the eligible participants approached for the study (0% nonparticipation rate). Owing to the low literacy levels among some patient participants and logistical constraints in remote field settings, transcripts were not returned to participants for formal member checking; however, preliminary findings were shared and discussed with local community health leaders to ensure contextual accuracy.

A detailed codebook outlining codes, categories, and thematic hierarchies for patients, MOs, and ASHAs or ANMs and nurses is provided in Multimedia Appendix 2.

### Ethical Considerations

This study is part of the telemedicine intervention project titled “Acceptance of Digital Medical Tools and Telemedicine in Hard-to-Reach Areas as Augmentation of Conventional Physical Clinics and Face-to-Face Consultation,” funded under grant IIRP-2023‐0000151. Ethical approval was obtained from the Institutional Ethics Committee of ICMR-NIIRNCD, Jodhpur, India (approval IEC-NIIRNCD/2023/DEC/FR/013; approved on December 22, 2023). The study was prospectively registered with the Clinical Trials Registry-India (CTRI/2024/10/076070). The study was conducted in accordance with the Declaration of Helsinki. Written informed consent was obtained from all participants prior to data collection. Participants were assured confidentiality and anonymity, and all identifying information was removed from transcripts before analysis. Participants did not receive financial compensation for their participation in the study; however, standard refreshments were offered during the interview sessions as a gesture of appreciation for their time.

## Results

### Overview

A total of 28 participants were included in the study, comprising 3 (10.7%) MOs, 10 (35.7%) nurses and ASHAs or ANMs, and 15 (53.6%) patients (3 from each of 5 villages). Data were collected through cadre-specific semistructured interviews, which were audio recorded and transcribed verbatim. Recruitment and data collection continued until thematic saturation was achieved within each participant group. Findings are presented cadre wise according to 3 participant categories. Participant characteristics and thematic tables are mentioned in Multimedia Appendix 3, and the COREQ checklist is presented in Checklist 2. An overview of key themes emerging from each cadre’s interviews is illustrated in Figures 1-3. The conceptual framework has been added to showcase the perspectives integrated across stakeholders in Figure 4. The final participant composition and characteristics are presented in Table 1.

**Figure 1.**
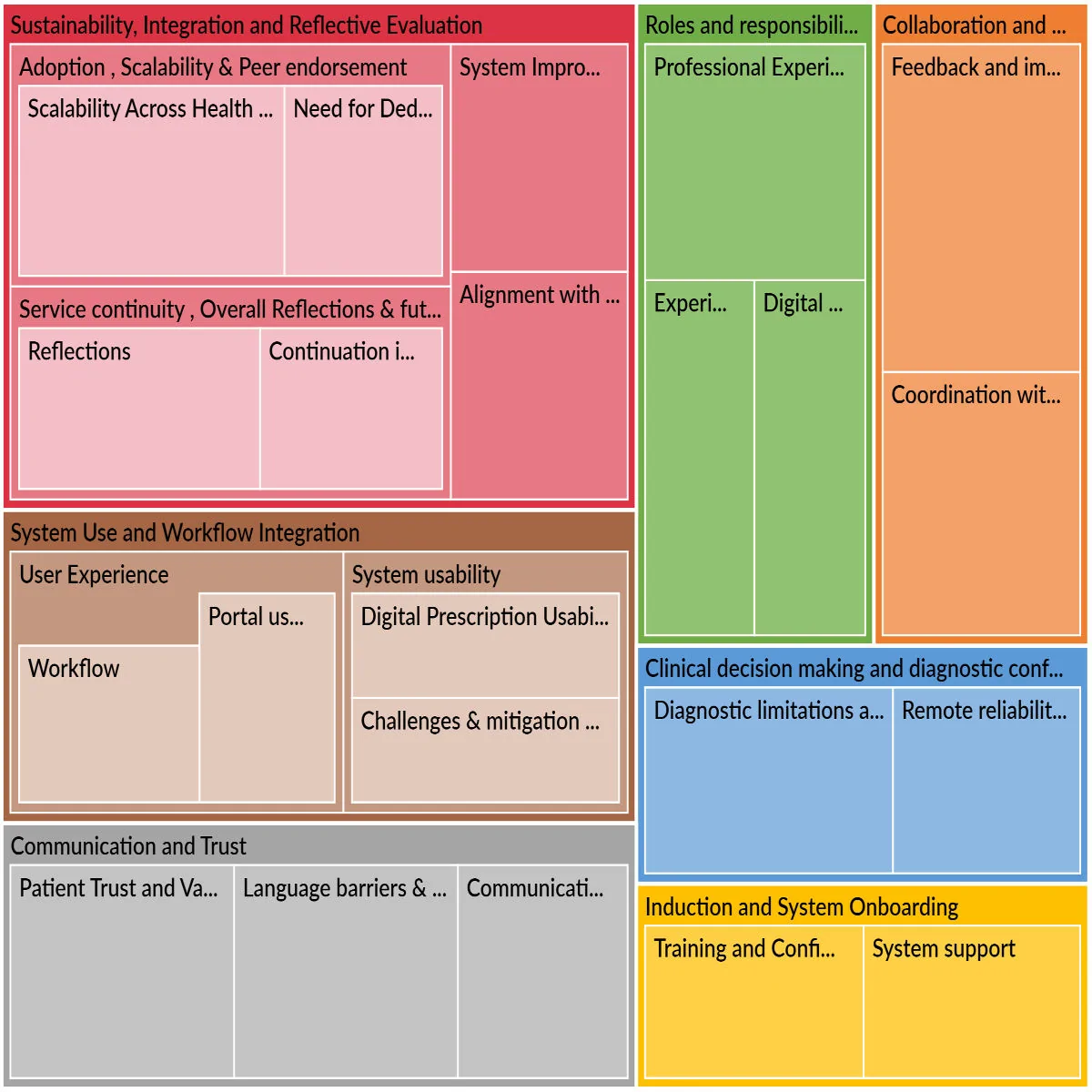
Thematic diagram of key themes and subthemes derived from interviews with medical officers.

**Figure 2.**
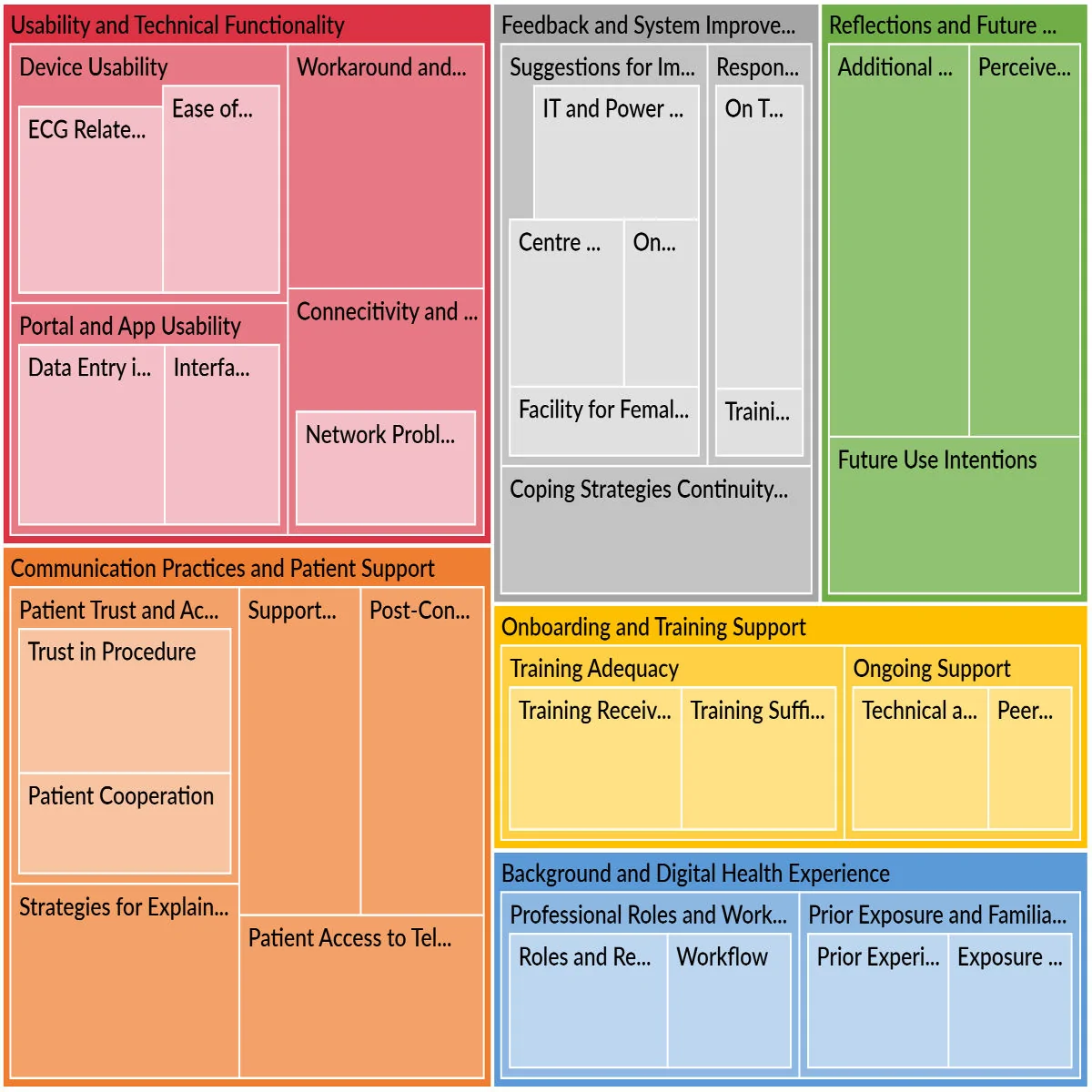
Thematic diagram illustrating key themes and subthemes derived from interviews with accredited social health activists or auxiliary nurse midwife and nursing officers.

**Figure 3.**
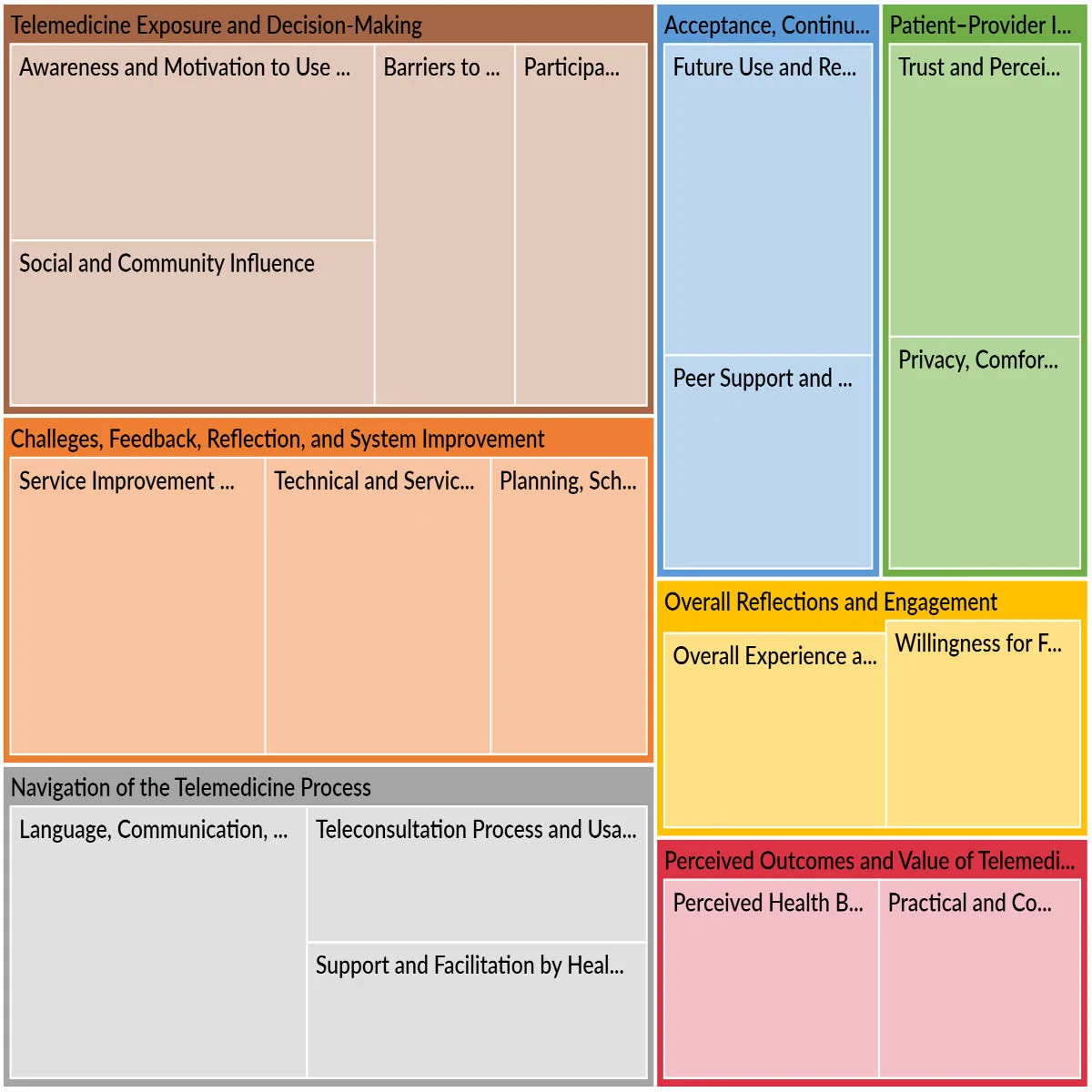
Thematic diagram illustrating key themes and subthemes derived from interviews with patients as beneficiaries of telemedicine intervention during the study period.

**Figure 4.**
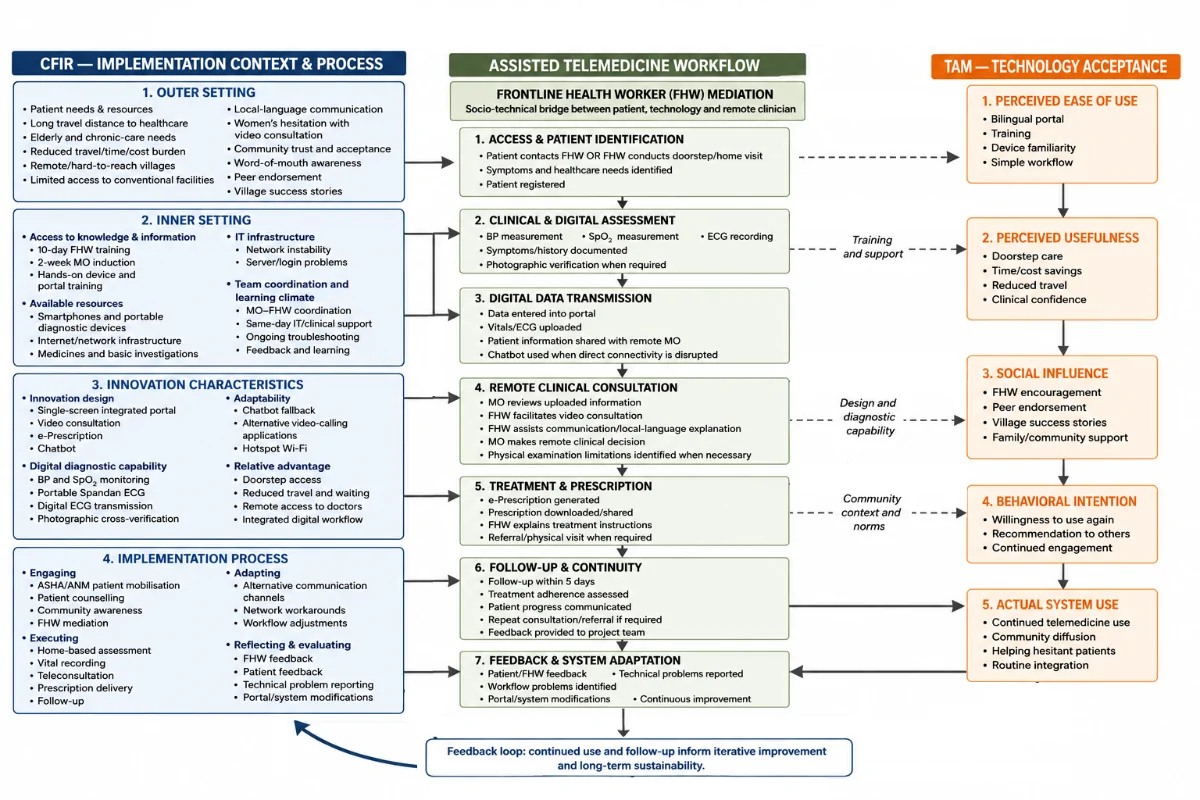
Conceptual framework of Consolidated Framework for Implementation Research and technology acceptance model in assisted telemedicine implementation and acceptance. ANM: auxiliary nurse midwife; ASHA: accredited social health activist; ECG: electrocardiogram; FHW: frontline health care worker; MO: medical officer; SpO_2_: oxygen saturation.

**Table 1.** Participant characteristics by cadre (N=28).

Characteristics	Medical officers (n=3)	Nurses (n=5)	ASHA^a^ or ANM^b^ (n=5)	Patients (n=15)	Total (n=28)
Age (years), mean (SD)	30.3 (2.1)	25.2 (1.5)	38.4 (7.3)	39.1 (12.8)	35.6 (12.2)
Sex, n (%)					
Male	3 (100)	3 (60)	0 (0)	8 (53.3)	14 (50)
Female	0 (0)	2 (40)	5 (100)	7 (46.7)	14 (50)
Religion, n (%)					
Hindu	3 (100)	5 (100)	5 (100)	14 (93.3)	27 (96.4)
Muslim	0 (0)	0 (0)	0 (0)	1 (6.7)	1 (3.6)
Marital status, n (%)					
Married	2 (66.7)	3 (60)	5 (100)	10 (66.7)	20 (71.4)
Unmarried	1 (33.3)	2 (40)	0 (0)	5 (33.3)	8 (28.6)
Languages spoken, n (%)^c^					
Marwari	3 (100)	5 (100)	5 (100)	15 (100)	28 (100)
Hindi	3 (100)	5 (100)	5 (100)	12 (80)	25 (89.3)
English	3 (100)	0 (0)	0 (0)	0 (0)	3 (10.7)
Education level, n (%)					
Uneducated	0 (0)	0 (0)	0 (0)	4 (26.7)	4 (14.3)
Primary (≤5th standard)	0 (0)	0 (0)	1 (20)	5 (33.3)	6 (21.4)
Secondary (10th-12th)	0 (0)	0 (0)	3 (60)	3 (20)	6 (21.4)
Graduate and above	0 (0)	3 (60)	1 (20)	3 (20)	7 (25)
Professional degree	3 (100)	2 (40)	0 (0)	0 (0)	5 (17.9)
Monthly income (INR), n (%)^d^	—^e^	—	—	—	15 (100)
<INR 5000 (US $52.21).	—	—	—	8 (53.3)	8 (53.3)
INR 5000 (US $52.21) to INR 10,000 (US $104.42)	—	—	—	1 (6.7)	1 (6.7)
INR 10,001 (US $104.42) to INR 20,000 (US $208.85)	—	—	—	5 (33.3)	5 (33.3)
>INR 20,000 (US $208.85)	—	—	—	1 (6.7)	1 (6.7)
Family type, n (%)^d^	—	—	—	—	15 (100)
Nuclear	—	—	—	3 (20)	3 (20)
Joint	—	—	—	12 (80)	12 (80)
Distance from health care center (km), n (%)^d^	—	—	—	—	15 (100)
<5	—	—	—	6 (40)	6 (40)
5‐10	—	—	—	4 (26.7)	4 (26.7)
10‐20	—	—	—	3 (20)	3 (20)
>20	—	—	—	2 (13.3)	2 (13.3)
Years of professional experience, n (%)^f^					
≤5	3 (100)	5 (100)	1 (20)	—	9 (69.2)
>5	0 (0)	0 (0)	4 (80)	—	4 (30.7)

a ASHA: accredited social health activist.

b ANM: auxiliary nurse midwife.

c Participants could speak more than 1 language; percentages for languages are calculated based on the total sample (n=28).

d Monthly income, family type, and distance from health care center are calculated only for patients (n=15).

e Not applicable.

f Professional experience is calculated only for health care workers (n=13), excluding patients.

### MOs’ Insights

#### Theme 1: Roles and Responsibilities—From Individual Readiness to Routine Telemedicine Practice

MOs described telemedicine adoption as a progressive process shaped by prior clinical experience, digital exposure, and evolving professional responsibilities. Their accounts reflected the CFIR domain of Individuals—Capability, as MOs drew on previous clinical training and project experience to operationalize remote care within their routine practice. One MO described his professional pathway and current responsibility within the telemedicine model:

*I have been working as a medical officer in the Telemedicine Project. Before this, I did my internship at my medical college, and after that, I joined here as a Medical Officer. My responsibility is to provide teleconsultation & I have 2 years of experience in the same project*.

[MO02]

Prior familiarity with digital health platforms also influenced the ease with which MOs transitioned to virtual consultations. In terms of the TAM construct of perceived ease of use, previous exposure to existing telemedicine systems reduced the learning burden associated with the new portal. One MO explained:

*I have used portals like e-Sanjeevani portals before this telemedicine project, so It has been comfortable enough for me to upload data, make video calls*.

[MO03]

At the same time, the introduction of new portable diagnostic devices required adaptation. This again reflected TAM’s perceived ease of use, as MOs compared earlier clinical tools with the simplified but unfamiliar portable equipment used in the project. One MO stated:

*Before the project, pulse oximeter, manual BP apparatus, and ECG machine of nine leads were part of my workflow, while in this project portable Spandan ECG machine with three leads has been a new experience for me*.

[MO03]

Together, these accounts suggest that MOs’ readiness for telemedicine was not uniform but was shaped by cumulative clinical experience, prior digital exposure, and gradual adaptation to new diagnostic and consultation workflows.

#### Theme 2: Training and System Onboarding—Building Confidence Through Structured Support

Structured training and ongoing system-level support were central to MOs’ confidence in delivering telemedicine. This aligned with the CFIR Inner Setting domain, particularly Access to Knowledge and Information, because formal induction training provided the knowledge required to use the portal, conduct video consultations, and generate e-prescriptions. One MO described the initial training process:

*Two weeks of induction training were provided on how to use the telemedicine portal, how to connect with nurses and patients through video calls, and how to generate e-prescriptions*.

[MO01]

In addition to formal training, MOs emphasized the importance of real-time collaboration with nursing and field staff. This reflected the CFIR construct of Available Resources within the Inner Setting because the presence of trained support staff during consultations made remote clinical workflows more manageable. One MO explained:

*The nursing staff also assisted in positioning the patient and sharing vital signs during the video call. This coordination made teleconsultation easier*.

[MO03]

MOs also reported that continuous technical and clinical support reduced early hesitation and enabled them to use the system more efficiently over time. This again highlighted the CFIR Inner Setting—Available Resources domain, as accessible support mechanisms helped convert initial uncertainty into routine use. One MO stated:

*Initially, we were not very confident about video consultations, but because technical and clinical support was always available, our hesitation reduced. Over time, it became more efficient and comfortable to use*.

[MO02]

These findings indicate that MOs’ adoption of telemedicine was supported not only by individual capability but also by structured onboarding, team-based assistance, and responsive technical support.

#### Theme 3: System Use and Workflow Integration—Platform Design and Adaptive Communication

Once MOs became familiar with the telemedicine system, platform design and adaptive communication strategies played an important role in integrating teleconsultations into routine clinical workflows. The centralized design of the portal was appreciated because it brought multiple consultation-related functions into a single interface. This reflected the CFIR construct of Innovation—Innovation Design, as a well-organized system reduced clinical friction and supported efficient decision-making. One MO explained:

*The telemedicine portal was well-designed and user-friendly. All patient details, vitals, doctor notes, video consultations, and e-prescriptions were available on a single screen, which reduced errors and saved time*.

[MO02]

However, network instability sometimes disrupted communication during consultations. MOs described using alternative communication mechanisms, such as the chatbot, when symptoms could not be clearly conveyed through video. This reflected the CFIR domain of Implementation Process—Tailoring Strategies because clinicians and field teams adapted the consultation process according to local connectivity constraints. One MO stated:

*There were network issues at patients’ side, because of which at times the symptoms were not clearly stated and understood, and then with the help of a chatbot, it was communicated*.

[MO03]

Technical difficulties such as server downtime and login problems were also reported, although MOs noted that these issues were generally resolved quickly. This again highlighted the importance of the CFIR Inner Setting—Information Technology Infrastructure, as the reliability of technical support influenced whether workflow disruptions became minor interruptions or major barriers to care. One MO explained:

*We faced server downtime, login issues due to poor network connectivity, and occasional portal crashes. However, these technical problems were resolved promptly*.

[MO01]

These accounts suggest that workflow continuity depended not only on the usability of the digital platform but also on the availability of fallback mechanisms and responsive technical support when connectivity or system problems occurred.

#### Theme 4: Clinical Decision-Making and Diagnostic Confidence—Remote Evidence and Clinical Boundaries

MOs reported that digital recording of vital signs and photographic verification strengthened their confidence in remotely obtained clinical information. This aligned with the CFIR construct of Innovation—Innovation Evidence Base because the ability to review entered vitals and cross-check photographic evidence enhanced trust in the quality of remote clinical data. One MO described the verification process:

*The field staff would send the data by typing in the vital signs and uploading photos of the digital devices on the chatbot, allowing us to cross-verify the data. If there was any issue with the readings or if the patient’s symptoms differed from those recorded, we would ask to recheck*.

[MO02]

At the same time, MOs recognized that telemedicine had clinical boundaries. Although remote consultations were sufficient for many routine conditions, certain clinical situations still required physical examination. This reflected the CFIR domain of Individuals—Capability, as MOs exercised professional judgment about when teleconsultation was appropriate and when in-person care was necessary. One MO stated:

*I was confident to treat patients as per the telemedicine guidelines, but certain cases of dermatological conditions, emergencies, and ANC visits needed in-person examination*.

[MO01]

Thus, telemedicine was perceived as a valuable model for routine primary care delivery, but MOs maintained clear clinical boundaries and referred patients to face-to-face examination when physical assessment was essential.

#### Theme 5: Communication and Trust—Frontline Mediation and Continuity of Care

MOs described patient acceptance as developing through continued communication, follow-up, and positive experiences with the service. This reflected the CFIR Inner Setting—Communication domain because sustained interaction between patients, frontline workers, and remote clinicians helped build trust in the telemedicine model. One MO reported:

*Patients reported high satisfaction with teleconsultation services and maintained regular contact with ASHA and ANM. Many continued follow-ups for chronic conditions and sought timely advice for acute illnesses*.

[MO01]

Communication during teleconsultations was also shaped by local language use and cultural expectations. This aligned with the CFIR Outer Setting—Local Attitudes domain because rural patients’ comfort with video-based care was influenced by language, gender norms, and familiarity with digital interaction. One MO explained:

*People from rural areas use local language, and a few patients, especially women, were shy or hesitant to speak on video*.

[MO03]

Continued information exchange between MOs and field workers supported follow-up and allowed clinicians to monitor patient progress. This reflected the CFIR construct of Innovation—Innovation Recipient Impact, as the telemedicine model influenced not only individual consultations but also ongoing care relationships. One MO stated:

*We took follow-ups when patients revisited for check-ups. Sometimes, field workers also updated us about the patient’s progress*.

[MO02]

These findings indicate that acceptance of telemedicine was supported not simply by the availability of remote consultation but by sustained interpersonal mediation and continuity between remote clinicians and community-based health workers.

#### Theme 6: Collaboration and Coordination—Team Learning and Feedback-Driven Improvement

MOs emphasized that telemedicine delivery depended on coordination among clinical, nursing, technical, and community teams. This reflected the CFIR Inner Setting—Learning Centeredness domain because collective preparation and shared understanding were important before the service was deployed in the field. One MO described this process:

*Before the field deployment of telemedicine services, we met, trained and explained he project details to all the ASHA, ANM, nursing staff, and peers*.

[MO02]

Feedback from clinical staff also contributed to iterative improvements in the telemedicine workflow. This aligned with the CFIR domain of Implementation Process—Reflecting and Evaluating because monthly review and practical feedback led to modifications that simplified the consultation process. One MO explained:

*Earlier, workflow required multiple systems for teleconsultations, but monthly feedback led to integration of video calls, chatbot, and e-prescription generation on a single screen*.

[MO02]

These accounts show that implementation was not a one-time deployment but an evolving process in which team coordination, training, and continuous feedback shaped the usability and effectiveness of the telemedicine system.

#### Theme 7: Sustainability, Integration, and Reflective Evaluation—Adaptation, Adoption, and Long-Term Value

MOs described several practical adaptations that helped sustain telemedicine delivery when digital infrastructure was unstable. This reflected the CFIR domain of Implementation Process—Adapting because clinicians and field teams developed workarounds to maintain service continuity. One MO stated:

*Network connectivity sometimes led to video call disruptions and portal lag, so we shifted to alternative modes such as video call through a hotspot Wi-Fi.*

[MO03]

MOs also viewed telemedicine as a valuable long-term addition to primary health care delivery. This aligned with the CFIR Inner Setting—Mission Alignment domain because telemedicine was seen as complementary to existing services and consistent with broader goals of improving access in remote areas. One MO explained:

*Telemedicine is a valuable long-term support tool with improvements in features such as chatbots and video calls to benefit patients in real-time*.

[MO01]

The commitment of MOs extended beyond routine working hours in some situations, reflecting both individual motivation and the adoptability of the model. This aligned with the CFIR construct of Implementation Outcome—Adoptability, as clinicians were willing to use personal devices when necessary to ensure continuity of care. One MO stated:

*Consultations were not done on weekends, but if needed, we used phones for consultations from our homes*.

[MO03]

MOs also acted as internal champions for telemedicine by discussing complex cases with colleagues and sharing positive experiences with peers. This again supported the CFIR domain of Implementation Outcome—Adoptability because professional endorsement and peer communication can strengthen broader acceptance of digital health innovations. One MO explained:

*We discussed critical cases and shared experiences to consult with our fellow specialists. I have shared the usefulness of telemedicine among rural populations with my peers*.

[MO01]

Finally, MOs reflected on the long-term benefits of telemedicine for patients, particularly older patients, while also identifying the need for privacy-sensitive physical spaces. This reflected the CFIR domain of Implementation Outcome—Reflecting and Evaluating because participants evaluated both the benefits of the service and the improvements required for equitable and sustainable implementation. One MO stated:

*Telemedicine enabled access to medical reports, e-prescriptions, and teleconsultations from home, reducing hospital visits, particularly for elderly patients. Additionally, the provision of a separate examination room for female patients or patients with sensitive conditions should be made*.

[MO01]

These reflections suggest that while MOs broadly endorsed the telemedicine model, they recognized that its long-term sustainability and equity depend on technological refinement, reliable infrastructure, peer support, and attention to culturally appropriate physical spaces at the point of care.

### Nurses’ Insights

#### Theme 1: Background and Digital Experience

Nurses and ASHA or ANM workers described their roles as extending beyond basic facilitation of teleconsultations to include patient assessment, vital sign measurement, digital data entry, coordination with remote doctors, patient education, and follow-up. This reflected the CFIR domain of Individuals—Capability, as frontline workers took on an expanded clinical and digital role within the assisted telemedicine model. One nurse described this end-to-end role:

*I accompanied ASHA and ANM workers to villages, shared contact details, responded to calls by visiting households, assessed patients' conditions, conducted teleconsultations, and shared e-prescriptions*.

[N04]

Similarly, an ASHA worker described simultaneously assessing patients, recording BP, SpO_₂_, and ECG, communicating findings to doctors, explaining prescriptions, and creating community awareness (ASHA01). These accounts indicate that the assisted telemedicine model fundamentally expanded the scope of frontline practice, positioning these workers as the primary clinical and digital interface between patients and remote doctors.

Prior clinical and technical exposure appeared to facilitate adaptation to the digital workflow. In terms of the TAM construct of perceived ease of use, existing familiarity with clinical monitoring equipment eased the transition to portable diagnostic tools, while structured hands-on training provided the skills required to perform the expanded role. A nurse noted previous experience with ventilators, patient monitoring systems, and 12-lead ECG, suggesting that existing familiarity with clinical monitoring equipment eased the transition to portable diagnostic tools (NO10). Structured onboarding further reinforced this readiness; one nurse described 10 days of practical training covering vital sign measurement on dummy patients, ECG operation, portal use, video consultations, chatbot functions, e-prescriptions, and monitoring doctor availability (NO04).

Together, these findings suggest that frontline workers’ initial readiness for telemedicine was shaped by prior clinical experience, digital familiarity, and the expanded professional responsibilities they assumed within the telemedicine workflow.

#### Theme 2: Onboarding and Training

Structured hands-on training provided the skills required to perform the expanded frontline role. This aligned with the CFIR Inner Setting—Access to Knowledge and Information domain because formal training equipped workers with the practical knowledge needed to operate devices, use the portal, and coordinate teleconsultations. One nurse described the training process:

*We received 10 days of training on taking BP, SpO₂, and ECG on a dummy patient, uploading data to the portal, conducting video calls with doctors, and logging into the nurse profile. We also learned to use the chatbot to enter patient details, upload medical reports, connect with doctors, receive e-prescriptions, and monitor doctors’ availability status*.

[N04]

Continued access to technical assistance further supported implementation. This reflected the CFIR Inner Setting—Available Resources domain, as responsive technical support helped frontline workers resolve operational problems quickly. One ASHA worker reported:

*We contacted the IT team of our department for any issues and usually received solutions the same day*.

[ASHA03]

Together, these findings suggest that frontline workers’ capacity to deliver telemedicine was built through a combination of prior clinical experience, structured practical training, and responsive post-deployment support.

#### Theme 3: Usability and Technical Functionality

Frontline workers generally found routine devices and the telemedicine portal manageable after training, although the portable ECG initially required additional familiarization. This aligned with the TAM construct of perceived ease of use, as workers’ confidence improved through training and repeated use. An ASHA worker explained:

*ECG was newer and more difficult at first, but after training, I understood it better*.

[ASHA04]

The portal and app were also considered accessible, with bilingual instructions in Hindi and English and clearly structured data entry fields facilitating use among workers with varying levels of digital familiarity. An ASHA worker stated:

*The portal and app were easy to understand with instructions in both Hindi and English and clear columns for details, so there was no problem using it*.

[ASHA03]

Connectivity remained an important operational constraint, particularly during video consultations. This reflected the CFIR Inner Setting—Information Technology Infrastructure domain because network limitations directly affected the reliability of real-time consultations. Workers reported using the chatbot and other communication channels when network availability was inadequate. As an ANM explained:

*Sometimes there was no network, then we used the chatbot or other alternatives for consultation*.

[ANM01]

Similarly, when the portal was unavailable, workers shifted consultations to alternative video calling apps and simultaneously reported technical failures to the IT team while maintaining direct communication with doctors. This reflected the CFIR domain of Implementation Process—Adapting, as frontline workers developed practical workarounds to maintain service continuity. One nurse stated:

*If the portal did not work, then we made the patient talk to the doctor through video call on another app*.

[N03]

Another nurse explained:

*We conveyed technical failure to the IT team and talked to the doctors directly on the video call*.

[N04]

Taken together, these accounts suggest that continuity of telemedicine depended not only on system usability but also on frontline workers’ ability to adapt workflows dynamically and access timely technical support when digital infrastructure failed.

#### Theme 4: Communication Practices and Patient Support

Nurses and community health workers played an important intermediary role in introducing telemedicine, addressing hesitation, and making remote consultations understandable to patients. This aligned with the CFIR Outer Setting—Local Conditions domain because frontline workers used existing community relationships to improve access and encourage use of the service. ASHA workers shared their phone numbers during awareness meetings and health camps so that patients could contact them directly when unwell. One ASHA worker reported:

*We shared our phone numbers during awareness meetings and camps, and patient called us directly whenever they fell sick so that we could visit them for teleconsultations*.

[ASHA02]

Local examples of patients who had benefited from telemedicine were used to build confidence among hesitant community members. This reflected the CFIR domain of Implementation Process—Engaging because trust was generated through local evidence and repeated interpersonal communication. One ASHA worker stated:

*We used examples of treated patients of the village itself to build trust*.

[ASHA01]

Continued counseling and motivation were also considered essential for patients accustomed to conventional face-to-face care. Another ASHA worker noted:

*With proper counselling and motivation, it is easier to make them believe and trust the telemedicine services*.

[ASHA02]

During consultations, frontline workers mediated linguistic and health literacy barriers by translating doctors’ instructions and explaining digital prescriptions. This reflected the CFIR domain of Implementation Process—Doing because frontline workers actively facilitated the consultation process and ensured that medical advice was understood. A nurse stated:

*During teleconsultation, if the patient did not understand the doctors’ instructions, then I explained the advice in their local language*.

[N02]

Another nurse described explaining prescription abbreviations and translating e-prescription instructions into the local language:

*We clarified instructions like BD (twice daily) or SOS (only when needed), and explained the e-prescription in local language to patients*.

[N03]

Gender and privacy concerns also influenced acceptance. This aligned with the CFIR construct of Innovation—Innovation Recipient Impact because patients’ acceptance of telemedicine was shaped by reassurance, confidentiality, and observed benefits. An ANM reported that some female patients initially hesitated about video consultations but became more accepting after reassurance about data confidentiality, discussion of other patients’ recovery, and explanation of the benefits of telemedicine:

*Female patients hesitated and doubted video calls, but after assurance of data confidentiality, citing fellow patients' recovery and explaining the benefits of telemedicine, they trusted and accepted the services*.

[ANM01]

The frontline role also extended beyond the initial teleconsultation to monitoring treatment adherence and assessing patient improvement. This reflected the TAM construct of perceived usefulness because follow-up reinforced the practical value of telemedicine for patients. A nurse described:

*During teleconsultation, if the patient is provided with a 7-day course, then we do a follow-up within 5 days to see whether they have taken the medicines on time, or any health improvement is noted or arrange another consultation*.

[N01]

Patients also returned with positive feedback and reported recovery, reinforcing frontline workers’ confidence in the service and validating the model’s clinical utility at the community level. An ASHA worker stated:

*Patients came back with positive feedback and reported recovery*.

[ASHA03]

These findings indicate that frontline workers functioned as clinical, linguistic, and cultural intermediaries, connecting the digital system with patients’ social, linguistic, and practical needs.

#### Theme 5: Feedback and System Improvement

Although frontline workers perceived clear benefits from telemedicine, they identified several conditions required for sustained implementation. This reflected the CFIR domain of Implementation Process—Assessing Context because participants highlighted gaps in medicines, physical infrastructure, connectivity, and devices that needed to be addressed to strengthen service delivery. One nurse summarized the importance of integrating medicines into the model:

*If the medicines were made available to us, we could support the patient in a better way*.

[N02]

An ASHA worker advocated for dedicated consultation spaces, noting:

*A proper place for teleconsultation should be set up with a medical facility where the patient can easily reach and be examined.*

[ASHA04]

An ANM further emphasized the need for technology adapted to rural connectivity constraints, stating:

*Low-internet-compatible devices and tablets are needed for smoother data entry and better video calls, along with simple video/user guides for troubleshooting*.

[ANM01]

Ongoing feedback from frontline workers additionally contributed to system improvement. This aligned with the CFIR domain of Implementation Process—Reflecting and Evaluating because technical and operational problems were reported, reviewed, and resolved during implementation. Participants described technical and operational problems being reported to the project team and resolved within a few days. One nurse stated:

*The issue reported to the team and that get solved next time or in 2-3 days*.

[N02]

Online training was also provided when additional support was required. One nurse explained:

*We were provided online training whenever needed to do things correctly in the field*.

[N05]

Coordination between frontline workers and remote doctors also provided an important safety mechanism during technical disruptions. This reflected the CFIR domain of Individuals—Capability, as clinicians and frontline workers used alternative communication methods to maintain care when the primary system failed. One nurse stated:

*When the portal was down, doctors supported us through alternate video calls, guiding patients with diagnosis and treatment so continuity of care was never disrupted*.

[N03]

Thus, continuity was supported by a combination of structured follow-up practices, interprofessional communication, responsive technical support, and iterative learning rather than by the digital platform alone.

#### Theme 6: Reflections and Future Engagement

Despite the constraints identified, frontline workers expressed strong support for continuation because telemedicine reduced travel, waiting time, and costs, particularly for older and geographically remote patients. This reflected the CFIR construct of Innovation—Relative Advantage and the TAM construct of perceived usefulness because frontline workers judged telemedicine to be beneficial compared with conventional care-seeking pathways. An ANM stated:

*Earlier we had to travel far and wait in queues; now telemedicine gives us treatment at home, saving time and money.*

[ANM01]

An ASHA worker similarly advocated for the service’s continuation, noting:

*Telemedicine should continue as it saves time and money and helps elderly and remote patients get treatment at home*.

[ASHA03]

At the same time, participants recognized that further improvements were necessary for long-term sustainability. This reflected the CFIR domain of Implementation Process—Reflecting and Evaluating because frontline workers evaluated both the benefits of the service and the system-level improvements required for continued delivery. One nurse summarized:

*Telemedicine gave timely care and relief at home, but better connectivity, medicines, and extended hours are needed.*

[N05]

These reflections suggest that while frontline workers strongly endorsed the telemedicine model, its long-term sustainability depends on addressing systemic gaps in physical infrastructure, medicine supply, digital connectivity, and service availability.

### Patients’ Insights

#### Theme 1: Telemedicine Addressed Important Barriers to Conventional Health Care Access

Patients described telemedicine primarily in relation to the practical difficulties they previously experienced when seeking conventional health care. This reflected the CFIR domain of the Outer Setting—Local Conditions, as participants reported long travel distances, transportation costs, accompanying family members, waiting times, and disruption of daily activities. One participant explained the logistical burden:

*With no nearby hospital, we had to travel 40 km or use private care, leading to an accompanying person, high costs and loss of time*.

[PT08]

Awareness of telemedicine was largely generated through ASHA workers, project staff, and community members, which aligned with the CFIR construct of Individuals—Motivation, as interpersonal networks drove the initial desire to try the service. As one participant described:

I *came to know about telemedicine through the villagers and the project staff, about the direct consultation with doctors on video call*.

[PT03]

Furthermore, positive experiences shared within villages encouraged its use, reflecting the TAM construct of social influence. Another participant noted:

*People in my village encouraged me to use telemedicine because of its benefits, but I haven't seen it used like this in nearby villages*.

[PT02]

These accounts indicate that initial engagement was driven not by institutional marketing but by organic, community-level diffusion through trusted personal networks. Additionally, patients’ baseline digital readiness was limited; reflecting the CFIR domain of Individuals—Capability, 1 patient recalled having seen a BP machine at the anganwadi but never at home (PT07), highlighting the absolute necessity of the assisted telemedicine model.

#### Theme 2: Local Health Workers Enabled Patients to Navigate the Digital Care Pathway

Patients did not generally experience telemedicine as a fully self-directed digital service. Instead, local health workers provided the practical bridge between patients and remote doctors. This reflected the CFIR domain of Implementation Process—Engaging, as frontline workers registered patients, recorded symptoms and vital signs, facilitated video consultations, and explained prescriptions. One participant described the structured workflow:

*Team visits homes daily to register patients, record symptoms, connect with doctors online, and note e-prescriptions*.

[PT15]

Another stated:

*The telemedicine staff and ASHA assisted us in teleconsultation with the doctor, explaining symptoms and e-prescription*.

[PT09]

This active facilitation compensated for the patients’ limited direct experience with digital devices. Patients’ confidence also developed through repeated exposure and communication in familiar language, which addressed the CFIR Outer Setting—Local Attitudes regarding cultural and linguistic comfort. One participant explained that the consultation language was easy to understand and that continued support could enable educated family members to assist with future use:

*The consultation language was easy to understand. With better systems and continued support, my educated family members can use it, and I can encourage them*.

[PT11]

Thus, patients’ engagement with telemedicine appeared to depend substantially on human mediation and the Innovation Design of the assisted delivery model rather than digital access alone.

#### Theme 3: Trust, Communication, and Privacy Shaped Acceptance of Remote Consultations

Patients described trust as developing through familiarity with local health workers, clear communication, and positive experiences with treatment. This aligned with the CFIR construct of Innovation—Innovation Evidence Base, as observable clinical effectiveness validated the remote care model. One participant stated:

*The team members have earned our trust and act as our trusted link to the doctors, especially because the treatment works*.

[PT07]

Privacy and confidentiality were also important for patients’ comfort during remote consultations. This reflected the CFIR Inner Setting—Recipient Centeredness, as respectful interactions and data protection ensured patient dignity in a close-knit community setting. One participant reported:

*My privacy was respected, and my health information was kept confidential*.

[PT01]

These accounts suggest that acceptance of telemedicine was influenced not only by access to a remote doctor but also by the quality of interpersonal mediation surrounding the consultation, including reassurance, understandable communication, and confidence that personal health information would remain private.

#### Theme 4: Convenience and Perceived Benefits Reinforced Continued Use

Patients consistently emphasized the practical value of receiving care closer to home. This strongly reflected the TAM construct of perceived usefulness, as telemedicine was perceived to reduce travel, time, financial expenditure, and disruption to daily activities. One participant reported:

*My health improved, and worries reduced as I do not have to go to the doctor physically and can get treatment at the doorstep*.

[PT10]

Another emphasized the comparative benefits:

*Telemedicine helps in avoiding long travelling distances, saves money, time and effort with sufficient care*.

[PT08]

These perceived advantages contributed to a strong TAM behavioral intention to continue using the service and to recommend it to others. One participant stated:

*We will definitely use these services because they come to our doorstep at lower cost and save our time and money*.

[PT12]

Furthermore, acceptance extended beyond individual users through organic peer support, reflecting actual system use and community-level diffusion. One patient reported actively supporting hesitant peers:

*I have helped women and elderly patients who were hesitant to speak on video calls by explaining and supporting them*.

[PT01]

This suggests that satisfied patients assumed informal advocacy roles that reinforced community-level adoption.

#### Theme 5: Technical and Service Limitations Remained Important Constraints

Despite generally positive experiences, patients identified limitations that restricted the completeness and reliability of telemedicine. Network instability sometimes resulted in interrupted audio and blurred video during consultations, highlighting constraints within the CFIR Inner Setting—Information Technology Infrastructure. One participant noted:

*Sometimes we faced network issues like unstable connectivity, voice breaks, and video blurring during video consultations*.

[PT09]

Participants also reported that medicines and certain diagnostic tests were not always available through the telemedicine service, requiring visits to government hospitals. This reflected gaps in local resource availability and the Outer Setting. One participant stated:

*They do not provide medicines or perform tests for dengue, malaria, blood infection and allergies, for which we have to visit nearby government hospitals, which is a disadvantage*.

[PT12]

Patients therefore suggested that the service would be more useful if medicines, injections, and basic investigations were available locally with trained staff:

*The service would improve if medicines, injections, and basic tests were available locally with trained staff*.

[PT08]

Importantly, patients also provided a balanced assessment of the service’s operational reliability, reflecting the CFIR Inner Setting—Work Infrastructure. One participant reported:

*We never faced any major planning or scheduling problems; only occasional delays happened when the network was poor, or doctors were busy*.

[PT05]

Patients further recognized the workload pressures on frontline workers and advocated for enhanced institutional support, noting:

*With proper training, enough staff, and support like medicines and tools, the health workers can manage the work smoothly and help everyone better*.

[PT09]

They also valued the responsiveness of the project team to feedback, an essential component of the CFIR Implementation Process—Reflecting and Evaluating, although quicker responses and follow-up were suggested as areas for improvement:

*The team listens to suggestions and responds well, but a quicker response and follow-up would help*.

[PT15]

#### Theme 6: Patients Viewed Telemedicine as a Useful Addition to Community Health Care

Overall, patients’ accounts reflected a positive assessment of telemedicine particularly because it brought medical consultation closer to their homes and reduced the logistical burden of conventional health care seeking. Engaging in the CFIR Implementation Process—Reflecting and Evaluating, one participant summarized this experience:

*Telemedicine has been very helpful for us as it gives good treatment in the village, saves time and money, avoids long travel, and makes us feel confident that care is available when we need it*.

[PT11]

At the same time, patients’ reflections indicate that continued engagement depended on maintaining the human support surrounding the technology and addressing practical limitations, such as connectivity, medicine availability, diagnostic services, staffing, and follow-up. Participants also expressed willingness to remain involved in future discussions and service improvement, reflecting the CFIR Implementation Outcome—Implementability and a sense of shared ownership. One participant stated:

*I am ready to talk again, and to help if needed in the future*.

[PT02]

This willingness to contribute suggests that patients had transitioned from passive recipients to active stakeholders in the telemedicine model.

## Discussion

### Principal Findings

A primary innovation of this study lies in the empirical validation of the assisted telemedicine model in hard-to-reach terrains. Unlike traditional direct-to-consumer telehealth models that assume a baseline of digital literacy, this study highlights a novel sociotechnical mediation framework where frontline workers (ASHAs, ANMs, and NOs) act as digital health navigators. By combining human cultural mediation with portable, specialized diagnostic tools (such as the Spandan ECG by Sunfox Technologies Pvt. Ltd.), this model overcomes the critical barriers of the digital divide and the lack of physical examination, offering a highly scalable blueprint for universal health coverage in resource-constrained settings. This study provides a comprehensive evaluation of telemedicine implementation within rural, hard-to-reach districts, specifically identifying the sociotechnical factors that facilitate digital adoption among underserved populations. By assessing the efficacy of an assisted model, using frontline health care workers and portable diagnostic tools for real-time vital screening, the findings suggest a potential shift in treatment-seeking behavior compared to traditional face-to-face consultations. This analysis offers valuable insights for optimizing digital health interventions.

### Sociotechnical Intermediation and Assisted Adoption Model

The persistent shortage of skilled health care providers in rural India remains a primary obstacle to achieving universal health coverage, with approximately 74% of medical practitioners serving only 28% of the population [15,16]. While the National Rural Health Mission has introduced financial incentives and contractual appointments, a significant gap remains in reaching isolated terrains. A distinguishing feature of this study is the identification of the assisted telemedicine model, where frontline workers act as essential technical and cultural mediators. Unlike previous models that rely on direct patient-to-provider interfaces, our findings reveal an increase in acceptance driven by ASHAs, ANMs, and nurses who bridge the digital divide for patients unfamiliar with screens. This is particularly crucial for women and older patients in hard-to-reach areas who may hesitate to consult male specialists or navigate complex portals [17]. By providing a human connection, the study demonstrates that acceptance is not merely a product of software functionality but of a structured human touch in a system that builds trust and overcomes cultural barriers [18,19].

### Clinical Efficacy of Digital Medical Tools and Decision-Making in Remote Care

A key contribution of this study is the evaluation of specialized digital tools, including the portable Spandan ECG (Sunfox Technologies Pvt. Ltd.) for real-time first-level cardiac screening, along with the measurement of vital parameters, such as BP, SpO_₂_, and heart rate. While telemedicine is often criticized for a lack of physical examination [20], our study reveals a robust workflow where field staff record vitals (BP, SpO_2_, and heart rate) and upload photographic evidence of digital devices via chatbots for clinician cross-verification. This protocol significantly enhances diagnostic confidence and data accuracy, a finding that moves beyond generic teleconsultation literature.

Despite these advancements, diagnostic limitations remain in dermatology and emergency care where physical palpation is irreplaceable. However, our findings align with those of large-scale health care systems, showing that sustained digital interaction can improve glycemic control and continuity of care for chronic conditions [21,22]. The efficacy of this model is therefore contingent on the robustness of rural digital infrastructure, such as BharatNet, and the hands-on training of health care workers to manage these sophisticated tools [23,24]. In such environments, the burden of managing chronic conditions shifts to these intermediaries, necessitating robust educational interventions to prevent implementation fatigue and ensure the quality of remote clinical evidence [25,26].

### Reconfiguring Treatment-Seeking Behavior and Health Equity

Our study provides empirical evidence of a fundamental shift in treatment-seeking behavior among rural populations. Traditionally, patients in remote Rajasthan or Maharashtra faced prohibitive logistical and financial barriers, often traveling up to 850 km for specialist care. By bringing health care to the “doorstep,” this study shows that telemedicine reduces attrition in traditional testing cascades and allows patients to reinvest the time saved into essential economic activities such as farming [27].

This study emphasizes that while telemedicine enhances equity and reduces financial hardship, its success is conditional. Trust building is a critical determinant; repeated technical failures or doctor unavailability can lead to a rapid loss of faith in the system. When functioning reliably, telemedicine acts as a peer-support and counseling platform, fostering treatment adherence. Ultimately, this research suggests that while digital health can bridge the health care divide, it requires addressing underlying gaps in connectivity, staffing, and trust to ensure long-term sustainability and systemic integration [28,29].

### Policy Implications for Scalable Digital Health Systems

The findings of this study underscore the need to shift health care policy from “provider-centric” to “mediated-digital” models. For telemedicine to be effectively integrated into the existing public health framework, policy interventions must prioritize formalizing the ASHA and ANM roles as “digital health navigators.” Furthermore, to sustain the positive shift in treatment-seeking behavior, future initiatives should focus on strengthening the rural digital infrastructure (BharatNet) and providing standardized, hands-on training for portable diagnostic tools.

### Strengths and Limitations

This study’s multistakeholder design provides a 360-degree view of the telemedicine ecosystem by integrating the perspectives of MOs, frontline health care workers (nurses and ASHAs or ANMs), and patients themselves. This approach enabled a holistic understanding of how the telemedicine model operates across different levels of the health care hierarchy. Furthermore, the use of the CFIR and TAM frameworks provided a robust theoretical foundation for moving beyond anecdotal evidence, enabling a structured analysis of how capability, motivation, and system design influence digital adoption in resource-limited settings. The inclusion of specific evaluations of portable diagnostic tools, such as the Spandan ECG (Sunfox Technologies Pvt. Ltd.), adds a layer of clinical relevance often missing from broader qualitative digital health studies.

While the study captures in-depth, real-world experiences of patients and frontline health care workers who were actively involved in telemedicine services, the findings are context specific to this implementation setting. The extended implementation period and inclusion of multiple stakeholder groups strengthen the relevance of the findings. Additionally, patients who opted to visit primary health Centerscenters despite the availability of telemedicine services were not included in this study.

### Conclusions

This study provides rich qualitative insights into the post-implementation delivery and use of telemedicine in remote areas of Rajasthan. Telemedicine was perceived as acceptable, useful, and supportive of continuity of care by both patients and frontline health care workers when embedded within existing primary health care systems. The shift in treatment-seeking behavior observed offers valuable evidence to support the further integration of digital health into the National Rural Health Mission.

Telemedicine should be viewed not as a secondary substitute for in-person care, but as a robust, complementary system that, when supported by trained mediators and reliable infrastructure, can significantly advance the goal of universal health coverage for India’s most vulnerable populations.

## Supplementary material

10.2196/93146 Multimedia Appendix 1 Interview guides used for in-depth qualitative interviews with study participants.

10.2196/93146 Multimedia Appendix 2 Codebooks.

10.2196/93146 Multimedia Appendix 3 Participant characteristics, study extraction, and thematic table derived from the qualitative interviews.

10.2196/93146 Checklist 1 iCHECK-DH guidelines and checklist for the reporting on digital health implementations.

10.2196/93146 Checklist 2 COREQ checklist.

## Abbreviations

ANM: auxiliary nurse midwife
ASHA: accredited social health activist
BP: blood pressure
CFIR: Consolidated Framework for Implementation Research
COREQ: Consolidated Criteria for Reporting Qualitative Research
ECG: electrocardiogram
iCHECK-DH: Guidelines and Checklist for the Reporting on Digital Health Implementations
ICMR: Indian Council of Medical Research
IDI: in-depth interview
MERN: MongoDB, Express, React, Node.js
MO: medical officer
NIIRNCD: National Institute for Implementation Research on Non-Communicable Diseases
NO: nursing officer
SpO2: oxygen saturation
TAM: technology acceptance model
