# Supplementary material for: Insights on Postimplementation and Delivery of Telemedicine in Remote Areas of Rajasthan: Qualitative Study
Source: JMIR Mhealth Uhealth. 2026 Oct 16;14:e93146. doi: 10.2196/93146 (PMC13675000; doi:10.2196/93146)
Supplement: Supplementary file 1 [file mhealth-v14-e93146-s001.docx]

**Semi-Structured Interview Guide for Patients**

**Objective: Understanding Community Use and Acceptance of Telemedicine Services in Rural India**

**Introduction**

Hello, and thank you for agreeing to participate.

We are conducting a study to understand how people experience and accept telemedicine services in rural areas. This conversation will help improve these services for others in your community. Your responses will be kept confidential and anonymous.

This interview will take about 30–45 minutes. With your permission, I’d like to audio-record our conversation. You can stop the interview or skip any question at any time.

Do you have any questions before we begin? Can I start recording?

[If yes]: Say “I will begin recording now.”

- [If not ok with recording interview]:
  - Ask “why not?” [since they agreed to this in the consent]
  - [Continue with the interview and take notes, but we may not be able to use responses in the analysis]

**Section A: Demographic Information (CFIR- Individuals: Capability)**

1. Can you tell me a bit about yourself? (Like your age, gender, marital status, occupation, and education level) And how long have you lived in this village?
2. Have you used any digital tools or devices before using telemedicine?

**Section B: Awareness and Access to Telemedicine (Framework: CFIR – Outer Setting / TAM- Social Influence)**

3. How did you first learn about the telemedicine services in your area? Probe: ASHA worker, word of mouth, poster, community meeting?
4. What made you decide to try this service? Probe: Convenience? Cost? Doctor availability? Previous experience?
5. What challenges, if any, did you face in accessing in-person healthcare before telemedicine was available? Probe: Transportation difficulties, long wait times, clinic distance, cost?
6. Did anyone in your family or community influence your decision to use telemedicine?
7. Have you seen or heard about telemedicine being used in other villages or regions?”

**Section C: Usability and Experience with the Service (Framework: TAM – Perceived Usefulness / CFIR – Intervention Characteristics)**

Transition: "Now that we’ve talked about how you learned about telemedicine, let’s discuss your experience using the service."

8. Can you describe your experience using the teleconsultation service? Probe: How did you connect to the doctor?
9. Was the process of communicating through the technology easy or difficult for you? Probe: Was any part of this process confusing to you?
10. Did someone assist you during the consultation (ASHA worker/ Project Nurse)? Probe: How helpful were they? Did you feel supported and understood?
11. Was the service available in your preferred language or dialect? (CFIR: Adaptability)
12. If the system changed (for example, a new app or platform), how confident would you feel using it? (CFIR: Self-Efficacy, Complexity)

**Section D: Acceptance and Usefulness of Telemedicine (Framework: TAM – Perceived Usefulness / Behavioural Intention)**

13. Did you feel that the teleconsultation helped improve your health or answer your concerns? Probe: Did the doctor understand your symptoms? Were you satisfied with the advice?
14. Would you use telemedicine again for future health concerns? Why or why not?
15. Do you trust the advice given through teleconsultation as much as advice from a face-to-face doctor visit?
16. Do you feel the doctor understood your local health context or background?
17. Have you recommended or would you recommend telemedicine to others?

**Section E: Perceived Benefits and Limitations (Framework: CFIR – Characteristics of Individuals / Outer Setting)**

18. What do you think are the advantages of telemedicine over regular doctor visits?

Probe: Has it saved you time, money, or effort in ways that matter to you?

19. What do you think are the disadvantages?
20. Was there any time when the teleconsultation did not work well for you? Probe: Technical failure, poor audio/video, doctor unavailable?

**Section F: Implementation Feedback (Framework: CFIR – Process / Inner Setting)**

21. In your opinion, how could the telemedicine service be improved? Probe: Timing, privacy, better explanations, clearer instructions?
22. Did you feel your privacy was respected during the consultation?
23. Are there any ways the healthcare workers (like ASHA) could be supported better in helping with telemedicine?
24. Did you feel your feedback or suggestions were welcomed by the health workers?
25. Were there any difficulties in scheduling or planning your consultation?
26. Would you feel comfortable helping others in your village learn to use telemedicine?

**Section G: Final Thoughts and Follow-Up (CFIR- Reflecting and Evaluating)**

27. Is there anything else you would like to share about your experience?
28. Would you be open to a follow-up interview or clarification call if needed?

**Closing Statement:**

Thank you again for your time and for sharing your experiences. Your input is incredibly valuable in helping us understand and improve telemedicine services in your community.

**Semi-Structured Interview Guide for Medical Officers (Doctors)**

**Objective: Understanding Provider Use and Experience with Telemedicine Services in Rural India**

Introduction

Hello, and thank you for agreeing to participate.

We are conducting a study to understand how doctors experience and utilize telemedicine services in rural areas. Your feedback will help improve the quality and delivery of digital health services for both patients and providers.

Your responses will remain confidential and anonymous. This interview will take about 30-45 minutes. With your permission, I’d like to audio-record our conversation. You may stop or skip any question at any time.

Do you have any questions before we begin? Can I start recording?

[If yes]: Say “I will begin recording now.”

- [If not ok with recording interview]:
  - Ask “why not?” [since they agreed to this in the consent]
  - [Continue with the interview and take notes, but we may not be able to use responses in the analysis]

**Section A: Background and Digital Experience (CFIR- Capability, TAM- Perceived Ease of Use)**

1. Can you briefly describe your current role, your responsibilities and years of experience as a medical officer?
2. How long have you been practising in this area and involved with this telemedicine project?
3. Have you used digital health tools (like EMR, e-prescriptions, or mobile apps) before this project? Probe: What kind of tools? How frequently?
4. How would you describe your comfort level with using digital platforms before this initiative?

**Section B: Onboarding and Adoption (CFIR – Inner Setting)**

5. What kind of training or orientation did you receive to begin using the platform? Probe: Did the training prepare you adequately for using the software and understanding workflows?
6. What support mechanisms (technical, clinical, or peer-based) are in place while using this system?

**Section C: System Use and Workflow Integration (CFIR – Innovation Design, Process- Tailoring Strategies)**

7. How would you describe your experience with the telemedicine portal interface? Probe: Was navigating the dashboard or entering patient vitals intuitive?
8. How well does the platform integrate with your usual clinical workflow? Probe: Does it save time or add extra steps compared to an in-person consultation?
9. How easy is it to generate and share e-prescriptions with patients?
10. Have you encountered any recurring challenges while using the system? Probe: Issues with login, patient record access, vitals display, sync errors, and updating records online?

**Section D: Clinical Decision-Making and Diagnostic Confidence (CFIR – Outer Setting, Inner Setting, Innovation)**

11. How reliable do you find the remote vitals (BP, ECG, SPO2, HR) shared by paramedics or ASHA workers?
12. Are there any diagnostic limitations you’ve faced because of not seeing the patient in person? Probe: Missing clinical signs, verbal cues, and physical examination issues?
13. Do you feel confident making treatment decisions through teleconsultation just based on available vitals data?
14. Have you noticed any types of cases where teleconsultation works particularly well or not well?

**Section E: Patient Interaction and Communication (CFIR – Outer Setting, Process- Reflecting and Evaluating)**

15. How do you typically communicate with patients during teleconsultations?
16. How effective do you feel the communication is with patients during teleconsultations? Probe: Do patients appear to understand and follow your advice and instructions given remotely?
17. Do patients express confidence in the teleconsultation, or do they ask for in-person validation?
18. Are language barriers or cultural expectations ever a challenge in remote consultations?

**Section F: Collaboration and System Support (CFIR – Networks & Communication / Process)**

19. How is your coordination with field workers like ASHAs, nurses, or technical support staff?
20. Do you feel your clinical inputs and suggestions for system improvements are acknowledged and implemented?
21. Are there opportunities for peer learning or sharing experiences with other doctors using the same platform?

**Section G: Reflections on Implementation and Sustainability (CFIR – Reflecting & Evaluating / Planning, Process- Adapting & Adoptability)**

22. What changes or improvements would you suggest for the platform or workflow?
23. How do you feel this model fits with long-term goals of improving rural healthcare?
24. Do you see yourself continuing to use this system after the pilot ends, if provided?
25. Would you recommend this telemedicine model to your peers in other regions?
26. How would you handle teleconsultations on weekdays or holidays?

**Section H: Final Thoughts and Follow-Up (CFIR-Reflecting and Evaluating)**

27. Is there anything else you would like to share about your experience with this telemedicine project?
28. Would you be open to a follow-up call or interview if clarification is needed?

**Closing Statement**

Thank you again for your time and for sharing your experience. Your insights are crucial in improving telemedicine services for doctors and patients alike.

**Semi-Structured Interview Guide for ASHA/ ANM Workers and Nurses**

**Objective: Understanding the Experience of Frontline Health Workers Using Telemedicine Services in Rural India**

Introduction

Hello, and thank you for agreeing to participate.

We are conducting a study to understand how frontline health workers like you experience and support telemedicine services in your community. This will help us improve these services for both workers and patients.

Your responses will remain confidential. The interview will take about 30–40 minutes. With your permission, I would like to audio-record our conversation. You may skip any question or stop at any time.

Do you have any questions before we begin? May I start the recording?

[If yes]: Say, “I will begin recording now.”

- [If not ok with recording interview]:
- Ask “why not?” [since they agreed to this in the consent]
- [Continue with the interview and take notes, but we may not be able to use responses in the analysis]

**Section A: Background and Digital Experience**

1. Can you tell me a little about yourself and your role as a health worker in this area?
2. What has been your role in the telemedicine project so far? Probe: What steps do you take when helping a patient use teleconsultation?
3. Before this project, had you used digital tools like smartphones, apps, or digital BP monitors? Probe: If yes, which ones and how often?
4. How comfortable did you feel using smart phones or digital tools when the project began?

**Section B: Training and Onboarding**

5. What kind of training did you receive to use the telemedicine system? Probe: Did it include how to use devices, log data, or start a call?
6. Was the training enough to help you feel ready to support patients? Probe: Did you need extra help or practice after training?
7. If you face a technical issue, who do you contact for help? Probe: Do you usually get quick responses?

**Section C: Using the Telemedicine System**

8. How easy or difficult is it to use the devices to measure vitals like BP, SPO2, ECG, etc.? Probe: Which devices are easiest or hardest for you?
9. How do you enter the data into the mobile app or portal? Probe: Any parts of the interface that confuse you?
10. Have you faced problems during video calls with the doctor? Probe:Poor audio, video not connecting, app crashing?
11. What do you do if the internet connection is weak or the app does not open?

**Section D: Communication and Patient Support**

12. How does a patient from the village contact you for illness treatment? Probe: Do you approach the patient or does the patient approach you?
13. How do you explain telemedicine to patients who are new to it? Probe: Do patients ever feel nervous or unsure about seeing the doctor on video?
14. How do you support the patient during the video consultation with the doctor? Probe: Do you help explain symptoms or the doctor's advice?
15. After the call, how do you ensure patients understand their prescription or next steps?
16. Do patients usually trust and follow the advice given by the doctor during teleconsultation?

**Section E: Feedback and System Improvement**

17. What challenges do you face while using the telemedicine system?
1. Probe: Device problems, patient issues, timing, existing healthcare system?
18. Have you ever shared suggestions to improve the process or app?
19. Were your suggestions listened to or acted upon? If yes, what?
20. What changes would make your work easier when helping patients through telemedicine?

**Section F: Final Thoughts and Follow-Up**

21. What do you think is the biggest benefit of telemedicine for your patients?
22. Would you want to continue using telemedicine in the future? Why or why not?
23. Is there anything else you would like to share about your experience?
24. Would you be willing to participate in a follow-up interview if needed?

**Closing Statement**

Thank you again for your time and your work in this program. Your experience is very important to help us improve this service for everyone in the community.
