# Supplementary material for: Insights on Postimplementation and Delivery of Telemedicine in Remote Areas of Rajasthan: Qualitative Study
Source: JMIR Mhealth Uhealth. 2026 Oct 16;14:e93146. doi: 10.2196/93146 (PMC13675000; doi:10.2196/93146)
Supplement: Supplementary file 2 [file mhealth-v14-e93146-s002.docx]

Patients Codebook

Codes

| Name | Files | References |
| --- | --- | --- |
| Acceptance, Continuity, and Community Diffusion | 15 | 37 |
| Future Use and Recommendation | 15 | 22 |
| Peer Support and Community Engagement | 15 | 15 |
| Challeges, Feedback, Reflection, and System Improvement | 15 | 65 |
| Planning, Scheduling, and Workforce Support | 15 | 16 |
| Service Improvement and Responsiveness | 15 | 26 |
| Technical and Service-Level Challenges | 15 | 23 |
| Navigation of the Telemedicine Process | 15 | 60 |
| Language, Communication, and Digital Confidence | 15 | 28 |
| Support and Facilitation by Health Workers | 15 | 16 |
| Teleconsultation Process and Usability | 15 | 16 |
| Overall Reflections and Engagement | 15 | 32 |
| Overall Experience and Meaning | 15 | 16 |
| Willingness for Follow-Up | 15 | 15 |
| Patient–Provider Interaction, Trust, and Privacy | 15 | 34 |
| Privacy, Comfort, and Dignity | 15 | 15 |
| Trust and Perceived Quality of Interaction | 15 | 19 |
| Perceived Outcomes and Value of Telemedicine | 15 | 31 |
| Perceived Health Benefits and Satisfaction | 15 | 16 |
| Practical and Comparative Benefits | 15 | 15 |
| Telemedicine Exposure and Decision-Making | 15 | 77 |
| Awareness and Motivation to Use Telemedicine | 15 | 24 |
| Barriers to Conventional Healthcare Access | 15 | 17 |
| Participant Context and Digital Readiness | 15 | 16 |
| Social and Community Influence | 15 | 20 |

ASHAs/ ANMs and Nursing Officer Codebook

Codes

| Name | Files | References |
| --- | --- | --- |
| Background and Digital Health Experience | 10 | 31 |
| Prior Exposure and Familiarity with Digital Health Tools | 10 | 15 |
| Exposure to Digital Devices | 7 | 7 |
| Prior Experience | 8 | 8 |
| Professional Roles and Workflow | 10 | 16 |
| Roles and Responsibilities | 9 | 9 |
| Workflow | 7 | 7 |
| Communication Practices and Patient Support | 10 | 58 |
| Patient Access to Telemedicine Services | 10 | 10 |
| Patient Trust and Acceptance | 10 | 17 |
| Patient Cooperation | 7 | 7 |
| Trust in Procedure | 10 | 10 |
| Post-Consult Follow-up | 10 | 10 |
| Strategies for Explaining Telemedicine to Patients | 10 | 11 |
| Support during Consultation | 10 | 10 |
| Feedback and System Improvement | 10 | 43 |
| Coping Strategies Continuity of Care | 10 | 10 |
| Responsiveness to Feedback | 10 | 11 |
| On Time Resolution | 9 | 9 |
| Training and Refresher | 2 | 2 |
| Suggestions for Improvements | 10 | 22 |
| Centre and Stay for Nurse | 6 | 6 |
| Facility for Female Patients | 4 | 4 |
| IT and Power Infrastructure | 5 | 7 |
| On-Site Medicine or First Aid Availability | 4 | 4 |
| Onboarding and Training Support | 10 | 32 |
| Ongoing Support | 10 | 13 |
| Peer Support and Collaboration | 5 | 5 |
| Technical and Supervisory Support | 8 | 8 |
| Training Adequacy | 10 | 19 |
| Training Received | 10 | 10 |
| Training Sufficiency and Refresher | 9 | 9 |
| Reflections and Future Engagement | 10 | 35 |
| Additional Reflections and Experience | 10 | 14 |
| Future Use Intentions | 10 | 10 |
| Perceived Benefits | 10 | 11 |
| Usability and Technical Functionality | 10 | 58 |
| Connecitivity and Technical Issues | 10 | 12 |
| Network Problem | 7 | 7 |
| Device Usability | 10 | 18 |
| Ease of Vital Devices | 8 | 8 |
| ECG Related Challenges | 9 | 9 |
| Portal and App Usability | 10 | 16 |
| Data Entry in Portal and App | 9 | 9 |
| Interface Understanding | 7 | 7 |
| Workaround and Adaptations | 10 | 12 |

Medical Officer Codebook

Codes

| Name | Files | References |
| --- | --- | --- |
| Clinical decision making and diagnostic confidence | 3 | 7 |
| Diagnostic limitations and decision making | 3 | 4 |
| Remote reliability of clinical evidence | 3 | 3 |
| Collaboration and Coordination | 3 | 9 |
| Coordination with system_level, team learning and peer | 3 | 4 |
| Feedback and implementation | 3 | 5 |
| Communication and Trust | 3 | 11 |
| Communication effectiveness | 3 | 3 |
| Language barriers & cultrural expectations | 3 | 4 |
| Patient Trust and Validation | 3 | 4 |
| Induction and System Onboarding | 3 | 6 |
| System support | 3 | 3 |
| Training and Confidence building | 3 | 3 |
| Roles and responsibilities | 3 | 10 |
| Digital tools exposure and comfort | 3 | 3 |
| Experience of Telemedicine | 3 | 3 |
| Professional Experience | 3 | 4 |
| Sustainability, Integration and Reflective Evaluation | 3 | 21 |
| Adoption , Scalability & Peer endorsement | 3 | 8 |
| Need for Dedicated After-Hours Support | 3 | 3 |
| Scalability Across Health Settings | 3 | 5 |
| Alignment with long term health goals | 3 | 3 |
| Service continuity , Overall Reflections & future engagement | 3 | 7 |
| Continuation intent post pilot | 3 | 3 |
| Reflections | 3 | 4 |
| System Improvement and Workflow Sustainability | 3 | 3 |
| System Use and Workflow Integration | 3 | 13 |
| System usability | 3 | 6 |
| Challenges & mitigation strategies | 3 | 3 |
| Digital Prescription Usability & Sharing | 3 | 3 |
| User Experience | 3 | 7 |
| Portal usability & Experience | 3 | 3 |
| Workflow | 3 | 3 |
