# Supplementary material for: Insights on Postimplementation and Delivery of Telemedicine in Remote Areas of Rajasthan: Qualitative Study
Source: JMIR Mhealth Uhealth. 2026 Oct 16;14:e93146. doi: 10.2196/93146 (PMC13675000; doi:10.2196/93146)
Supplement: Supplementary file 3 [file mhealth-v14-e93146-s003.docx]

**Supplementary File**

**Table S1. Sample size of study participants**

| **Participant type** | **Number of IDIs** | **Associated health facility** |
| --- | --- | --- |
| Medical Officer (MO) | 3 | Project headquarters / PHC-level facility |
| Nursing Officer (NO) | 5 | Village-level field sites |
| ASHA / ANM | 5 | Village-level field sites |
| Patients | 15 | Resident of villages and beneficiaries of telemedicine service in intervention villages |
| **Total** | **28** | — |

**Table S2. Demographic characteristics of participants**

| **S. No.** | **Code** | **Role** | **Location** | **Age** | **Gender** | **Years in Practice** | **Education Level** | **Timing** |
| --- | --- | --- | --- | --- | --- | --- | --- | --- |
| 1 | MO01 | Medical Officer | Urban | 31 | M | 04 | M.B.B.S. | 26 min |
| 2 | MO02 | Medical Officer | Urban | 28 | M | 01 | M.B.B.S. | 41 min |
| 3 | MO03 | Medical Officer | PHC-I | 32 | M | 05 | M.B.B.S. | 20 min |
| 4 | NO01 | Nursing Officer | Village-I | 26 | M | 2.5 | G.N.M. Nursing | 37 min |
| 5 | NO02 | Nursing Officer | Village-II | 27 | M | 02 | B.Sc. Nursing | 30 min |
| 6 | NO03 | Nursing Officer | Village-III | 23 | F | 01 | A.N.M. Nursing | 23 min |
| 7 | NO04 | Nursing Officer | Village-IV | 25 | F | 01 | A.N.M. Nursing | 26 min |
| 8 | NO05 | Nursing Officer | Village-V | 25 | M | - | B.Sc. Nursing | 23 min |
| 9 | ANM01 | Auxiliary Nurse Midwifery | Village-IV | 45 | F | 25 | G.N.M. Nursing | 20 min |
| 10 | ASHA01 | Accredited Social Health Activist | Village-I | 29 | F | 06 | 12^th^ | 27 min |
| 11 | ASHA02 | Accredited Social Health Activist | Village-II | 37 | F | 15 | 12^th^ | 32 min |
| 12 | ASHA03 | Accredited Social Health Activist | Village-III | 48 | F | 04 | 5^th^ | 32 min |
| 13 | ASHA04 | Accredited Social Health Activist | Village-V | 33 | F | 5.5 | B.Ed. (M.A.) | 32 min |
| 14 | PT01 | Patient-01 | Village-I | 44 | M | NA | 5^th^ | 25 min |
| 15 | PT02 | Patient-02 | Village-I | 62 | M | NA | Uneducated | 43 min |
| 16 | PT03 | Patient-03 | Village-I | 30 | M | NA | Graduate | 32 min |
| 17 | PT04 | Patient-04 | Village-II | 35 | F | NA | Uneducated | 45 min |
| 18 | PT05 | Patient-05 | Village-II | 50 | F | NA | Uneducated | 35 min |
| 19 | PT06 | Patient-06 | Village-II | 24 | M | NA | Graduate | 43 min |
| 20 | PT07 | Patient-07 | Village-III | 28 | F | NA | 10^th^ | 43 min |
| 21 | PT08 | Patient-08 | Village-III | 65 | M | NA | 5^th^ | 38 min |
| 22 | PT09 | Patient-09 | Village-III | 45 | F | NA | Uneducated | 34 min |
| 23 | PT10 | Patient-10 | Villages-IV | 23 | F | NA | Graduate | 39 min |
| 24 | PT11 | Patient-11 | Villages-IV | 37 | M | NA | 5^th^ | 25 min |
| 25 | PT12 | Patient-12 | Village-IV | 50 | M | NA | 5^th^ | 46 min |
| 26 | PT13 | Patient-13 | Village-V | 19 | F | NA | 10^th^ | 32 min |
| 27 | PT14 | Patient-14 | Village-V | 25 | M | NA | Graduate | 22 min |
| 28 | PT15 | Patient-15 | Village-V | 50 | F | NA | 10^th^ | 35 min |

**Table S3. Thematic Analysis Coding for Medical Officers, ASHAs/ ANMs, Nursing Officers and Patients**

| **Cadre Classification** | **Theme** | **Sub-theme** | **Code name (with construct)** | **Research Questions** | **Files** | **References** |
| --- | --- | --- | --- | --- | --- | --- |
| Medical Officers | Role and Responsibilities | Professional experience | Individuals- Capability (CFIR) | "Can you briefly describe your current role, your responsibilities and years of experience as a medical officer?" | 3 | 4 |
|  |  | Experience of Telemedicine | Individuals- Capability (CFIR) | "How long have you been practising in this area and involved with this telemedicine project?" | 3 | 3 |
|  |  | Digital tools exposure & comfort | Perceived Ease of Use (TAM) | "Have you used digital health tools (like EMR, e-prescriptions, or mobile apps) before this project? How would you describe your comfort level with using digital platforms before this initiative? " | 3 | 3 |
|  | Induction and system onboarding | Training and confidence building | Inner Setting - Access to Knowledge and Information | "What kind of training or orientation did you receive to begin using the platform?" | 3 | 3 |
|  |  | System support | Inner Setting - Available Resources & Support (CFIR) | "What support mechanisms (technical, clinical, or peer-based) are in place while using this system?" | 3 | 3 |
|  | System use and workflow integration | User experience | Innovation - Innovation Design (CFIR) | "How would you describe your experience with the telemedicine portal interface?"  "How well does the platform integrate with your usual clinical workflow?" | 3 | 7 |
|  |  | System usability | Implementation Process - Tailoring Strategies (CFIR) | "How easy is it to generate and share e-prescriptions with patients?"  " Have you encountered any recurring challenges while using the system?" | 3 | 6 |
|  | Clinical decision making and diagnostic confidence | Remote reliability of clinical evidence | Individuals - Capability (CFIR) | How reliable do you find the remote vitals (BP, ECG, SPO2, HR) shared by paramedics or ASHA workers | 3 | 3 |
|  |  | Diagnostic limitations and decision making | Innovation - Innovation Evidence Base (CFIR) | "Are there any diagnostic limitations you’ve faced because of not seeing the patient in person? Do you feel confident making treatment decisions through teleconsultation just based on available vitals data?" Have you noticed any types of cases where teleconsultation works particularly well or not well ?" | 3 | 4 |
|  | Communication and trust | Patient trust and validation | Inner Setting - Communications (CFIR) | "Do patients express confidence in the teleconsultation, or do they ask for in-person validation?" | 3 | 4 |
|  |  | Language barrier and cultural expectations | Outer Setting – Local attitudes (CFIR) | "How do you typically communicate with patients during teleconsultations?" "Are language barriers or cultural expectations ever a challenge in remote consultations?" | 3 | 4 |
|  |  | Communication effectiveness | Innovation - Innovation Recipient Impact (CFIR) | "How effective do you feel the communication is with patients during teleconsultations?" | 3 | 3 |
|  | Collaboration and coordination | coordination with system-level, team-learning and peers | Inner Setting - Learning Centredness (CFIR) | "How is your coordination with field workers like ASHAs, nurses, or technical support staff?" "Are there opportunities for peer learning or sharing experiences with other doctors using the same platform?" | 3 | 4 |
|  |  | feedback mechanism and implementation | Process – Reflecting & Evaluating (CFIR) | "Do you feel your clinical inputs and suggestions for system improvements are acknowledged and implemented?" | 3 | 5 |
|  | Sustainability, Integration and Reflective Evaluation | System Improvement and Workflow Sustainability | Implementation Process – Adapting (CFIR) | "What changes or improvements would you suggest for the platform or workflow?" | 3 | 3 |
|  |  | Alignment with long term Health goals | Inner Setting- Mission Alignment (CFIR) | "How do you feel this model fits with long-term goals of improving rural healthcare?" | 3 | 3 |
|  |  | Adoption, Scalability & Peer endorsement | Implementation Outcomes – Adoptability (CFIR) | "Would you recommend this telemedicine model to your peers in other regions?"  "How would you handle teleconsultations on weekdays or holidays?" | 3 | 8 |
|  |  | Service continuity, Overall Reflections & future engagement | Implementation Outcome - Reflecting and Evaluating (CFIR) | " Do you see yourself continuing to use this system after the pilot ends, if provided? "  " Is there anything else you would like to share about your experience with this telemedicine project?" | 3 | 7 |
| Nursing Officer and ASHAs/ ANMs | Background & Digital Experience | Professional Role & Workflow | Individuals – Capability (CFIR) | “Can you tell me something about yourself and your role as a healthcare worker?”  “How long have you been practising in this area and involved with this telemedicine project?” | 10 | 16 |
|  |  | Prior Exposure and familiarity to Digital Tools | Perceived Ease of Use (TAM) | “Before using telemedicine, did you use smartphones, apps, or medical devices?”  “How comfortable were you using these digital devices before this initiative?” | 10 | 15 |
|  | Onboarding & Training | Training Adequacy | Inner Setting - Access to Knowledge & Information (CFIR) | “What kind of training did you receive to use the telemedicine system? Did it include devices, log data, or calls?”  “Was the training enough to help you feel ready to support patients? Did you need extra help?” | 10 | 19 |
|  |  | Ongoing Support | Inner Setting - Available Resources (CFIR) | “If you face a technical issue, who do you contact for help? Do you usually get quick responses?”  “Did you seek help from peers while learning telemedicine tasks?” | 10 | 13 |
|  | Usability and Technical Functionality | Device Usability | Perceived Ease of Use (TAM) | “How easy or difficult is it to use BP, SPO2, and ECG?  “Which devices are easiest or hardest?”  “Did you face any challenges using ECG?” | 10 | 18 |
|  |  | Portal/App Usability | Perceived Ease of Use (TAM) | “How do you enter the data into the mobile app/portal? Any confusing parts?”  “Was there any interface or part of the portal that was confusing?” | 10 | 16 |
|  |  | Connectivity & Technical Issues | Inner Setting - Information Technology Infrastructure (CFIR) | “Have you faced problems during video calls with the doctor?” | 10 | 12 |
|  |  | Workaround and Adaptations | Implementation Process - Adapting (CFIR) | “What do you do if the internet is weak or the app doesn’t open?” | 10 | 12 |
|  | Communication Practices & Patient Support | Patient Access to Telemedicine Services | Outer Setting - Local Conditions (CFIR) | “How does a patient from the village contact you for illness treatment?”  “Do you approach them or do they approach you?” | 10 | 10 |
|  |  | Strategies for Explaining Telemedicine to Patients | Implementation Process - Engaging (CFIR) | “How do you explain telemedicine to patients who are new to it?”  “Do patients feel nervous or unsure?” | 10 | 11 |
|  |  | Support During Consultation | Implementation Process - Doing (CFIR) | “How do you support patients during the video consultation with the doctor? Do you explain symptoms or the doctor’s advice?” | 10 | 10 |
|  |  | Post-Consult Follow-Up | Perceived Usefulness (TAM) | “After the call, how do you ensure patients understand their prescription or next steps? How did you make sure patients follow the doctor’s advice?” | 10 | 10 |
|  |  | Patient Trust & Acceptance | Innovation Outcomes - Innovation Recipient Impact (CFIR) | “Do patients usually trust and follow the advice given by the doctor during teleconsultation?”  “Were female or male patients more comfortable during teleconsultations? Did cultural expectations affect use?” | 10 | 17 |
|  | Feedback and System Improvement | Suggestions for Improvement | Implementation Process- Assessing Context (CFIR) | “Have you ever shared suggestions to improve the process or app? Were your suggestions listened to?” | 10 | 22 |
|  |  | Responsiveness to feedback | Implementation Process- Reflecting & Evaluating (CFIR) | “Were your suggestions acted upon? If yes, what changes were made?” | 10 | 11 |
|  |  | Coping strategies and continuity of care | Individual - Capability (CFIR) | “What changes would make your work easier when helping patients?” | 10 | 10 |
|  | Reflections & Future Engagement | Perceived Benefits | Innovation- Innovation relative (CFIR) | “What do you think is the biggest benefit of telemedicine for your patients?” | 10 | 11 |
|  |  | Future Use Intentions | Implementation Outcome - Adaptability (CFIR) | “Would you want to continue using telemedicine in the future? Why or why not?” | 10 | 10 |
|  |  | Additional Reflections | Implementation Process - Reflecting and Evaluating (CFIR) | “Is there anything else you’d like to share about your experience?” | 10 | 14 |
| Patients (Telemedicine Beneficiary) | Telemedicine Exposure and Decision-Making | Participant Context and Digital Readiness | Individuals-Capability (CFIR) | Can you tell me a bit about yourself (age, gender, marital status, occupation, education, and how long you’ve lived in this village)?; Have you used any digital tools or devices before using telemedicine? | 15 | 16 |
|  |  | Awareness and Motivation to Use Telemedicine | Individuals-Motivation (CFIR) | How did you first learn about the telemedicine services in your area? What made you decide to try this service? | 15 | 24 |
|  |  | Social and Community Influence | Social Influence (TAM) | Did anyone in your family or community influence your decision to use telemedicine? Have you seen or heard about telemedicine being used in other villages or regions? | 15 | 20 |
|  |  | Barriers to Conventional Healthcare Access | Outer Setting- Local Conditions (CFIR) | What challenges, if any, did you face in accessing in-person healthcare before telemedicine was available? | 15 | 17 |
|  | Navigation of the Telemedicine Process | Teleconsultation Process and Usability | Innovation - Innovation design (CFIR) | Can you describe your experience using the teleconsultation service? Was the process of communicating through the technology easy or difficult for you? | 15 | 16 |
|  |  | Support and Facilitation by Health Workers | Implementation - Engaging (CFIR) | Did someone assist you during the consultation (ASHA worker/Project Nurse)? How helpful were they? | 15 | 16 |
|  |  | Language, Communication, and Digital Confidence | Outer Setting- Local Attitude (CFIR) | Was the service available in your preferred language or dialect? If the system changed (for example, a new app or platform), how confident would you feel using it? | 15 | 28 |
|  | Patient–Provider Interaction, Trust, and Privacy | Trust and Perceived Quality of Interaction | Innovation- Innovation Evidence Base | Do you trust the advice given through teleconsultation as much as advice from a face-to-face doctor visit? Do you feel the doctor understood your local health context or background? | 15 | 19 |
|  |  | Privacy, Comfort, and Dignity | Inner Setting - Recipient Centredness) | Did you feel your privacy was respected during the consultation? | 15 | 15 |
|  | Perceived Outcomes and Value of Telemedicine | Perceived Health Benefits and Satisfaction | Perceived Usefulness (TAM) | Did you feel that the teleconsultation helped improve your health or answer your concerns? | 15 | 16 |
|  |  | Practical and Comparative Benefits | Perceived Usefulness (TAM) | What do you think are the advantages of telemedicine over regular doctor visits? | 15 | 15 |
|  | Acceptance, Continuity, and Community Diffusion | Future Use and Recommendation | Behavioural Intention (TAM) | Would you use telemedicine again for future health concerns? Why or why not?; Have you recommended or would you recommend telemedicine to others? | 15 | 22 |
|  |  | Peer Support and Community Engagement | Actual System Use (TAM) | Would you feel comfortable helping others in your village learn to use telemedicine? | 15 | 15 |
|  | Challenges, Feedback, Reflection, and System Improvement | Technical and Service-Level Challenges | Inner Setting - Information Technology and Infrastructure (CFIR) | What do you think are the disadvantages of telemedicine? Was there any time when the teleconsultation did not work well for you? | 15 | 23 |
|  |  | Service Improvement and Responsiveness | Implementation Process - Reflecting and Evaluating (CFIR) | In your opinion, how could the telemedicine service be improved? Did you feel your feedback or suggestions were welcomed by the health workers? | 15 | 26 |
|  |  | Planning, Scheduling, and Workforce Support | Inner setting - Work Infrastructure (CFIR) | Are there any ways the healthcare workers (like ASHA) could be supported better in helping with telemedicine? Were there any difficulties in scheduling or planning your consultation? | 15 | 16 |
|  | Overall Reflections and Engagement | Overall Experience and Meaning | Implementation Process - Reflecting and Evaluating (CFIR) | Is there anything else you would like to share about your experience? | 15 | 32 |
|  |  | Willingness for Follow-Up | Implementation Outcome - Implementability (CFIR) | Would you be open to a follow-up interview or clarification call if needed? | 15 | 15 |
